# Kek-6: A truncated-Trk-like receptor for *Drosophila* neurotrophin 2 regulates structural synaptic plasticity

**DOI:** 10.1371/journal.pgen.1006968

**Published:** 2017-08-28

**Authors:** Suzana Ulian-Benitez, Simon Bishop, Istvan Foldi, Jill Wentzell, Chinenye Okenwa, Manuel G. Forero, Bangfu Zhu, Marta Moreira, Mark Phizacklea, Graham McIlroy, Guiyi Li, Nicholas J. Gay, Alicia Hidalgo

**Affiliations:** 1 NeuroDevelopment Group, School of Biosciences, University of Birmingham, Edgbaston, Birmingham, United Kingdom; 2 Grupo D+Tec, Universidad de Ibagué, Ibagué, Tolima, Colombia; 3 Department of Biochemistry, University of Cambridge, Cambridge, United Kingdom; Francis Crick Institute, UNITED KINGDOM

## Abstract

Neurotrophism, structural plasticity, learning and long-term memory in mammals critically depend on neurotrophins binding Trk receptors to activate tyrosine kinase (TyrK) signaling, but *Drosophila* lacks full-length Trks, raising the question of how these processes occur in the fly. Paradoxically, truncated Trk isoforms lacking the TyrK predominate in the adult human brain, but whether they have neuronal functions independently of full-length Trks is unknown. *Drosophila* has TyrK-less Trk-family receptors, encoded by the *kekkon (kek)* genes, suggesting that evolutionarily conserved functions for this receptor class may exist. Here, we asked whether Keks function together with Drosophila neurotrophins (DNTs) at the larval glutamatergic neuromuscular junction (NMJ). We tested the eleven LRR and Ig-containing (LIG) proteins encoded in the *Drosophila* genome for expression in the central nervous system (CNS) and potential interaction with DNTs. Kek-6 is expressed in the CNS, interacts genetically with DNTs and can bind DNT2 in signaling assays and co-immunoprecipitations. Ligand binding is promiscuous, as Kek-6 can also bind DNT1, and Kek-2 and Kek-5 can also bind DNT2. In vivo, Kek-6 is found presynaptically in motoneurons, and DNT2 is produced by the muscle to function as a retrograde factor at the NMJ. Kek-6 and DNT2 regulate NMJ growth and synaptic structure. Evidence indicates that Kek-6 does not antagonise the alternative DNT2 receptor Toll-6. Instead, Kek-6 and Toll-6 interact physically, and together regulate structural synaptic plasticity and homeostasis. Using pull-down assays, we identified and validated CaMKII and VAP33A as intracellular partners of Kek-6, and show that they regulate NMJ growth and active zone formation downstream of DNT2 and Kek-6. The synaptic functions of Kek-6 could be evolutionarily conserved. This raises the intriguing possibility that a novel mechanism of structural synaptic plasticity involving truncated Trk-family receptors independently of TyrK signaling may also operate in the human brain.

## Author summary

A long-standing paradox had been to explain how brain structural plasticity, learning and long-term memory might occur in Drosophila in the absence of canonical Trk receptors for neurotrophin (NT) ligands. NTs link structure and function in the brain enabling adjustments in cell number, dendritic, axonal and synaptic patterns, in response to neuronal activity. These events are essential for brain development, learning and long-term memory, and are thought to depend on the tyrosine-kinase function of the NT Trk receptors. However, paradoxically, the most abundant Trk isoforms in the adult human brain lack the tyrosine kinase, and their neuronal function is unknown. Remarkably, Drosophila has kinase-less receptors of the Trk family encoded by the *kekkon (kek)* genes, suggesting that deep evolutionary functional conservation for this receptor class could be unveiled. Here, we show that Kek-6 is a receptor for Drosophila neurotrophin 2 (DNT2) that regulates structural synaptic plasticity via CaMKII and VAP33A. The latter are well-known factors regulating synaptic structure and plasticity and vesicle release. Furthemore, Kek-6 cooperates with the alternative DNT2 receptor Toll-6, and their concerted functions are required to regulate structural homeostasis at the NMJ. Our findings suggest that in mammals truncated Trk-family receptors could also have synaptic functions in neurons independently of Tyrosine kinase signaling. This might reveal a novel mechanism of brain plasticity, with important implications for understanding also the human brain, in health and disease.

## Introduction

Brain plasticity, neurotrophism in development, structural and synaptic plasticity during learning and long-term memory in humans critically depend on the receptor TrkB binding its neurotrophin (NT) ligand BDNF [1,2]. During development, NTs and Trks regulate neuronal number and connectivity; subsequently BDNF and TrkB establish a reinforcing positive feedback loop promoting synaptic potentiation, and regulate the dynamic generation and elimination of synaptic boutons and dendritic spines in response to activity [1,3–5]. Thus, NTs and Trks are fundamental to linking structure and function in the brain, and this is thought to depend mostly on the tyrosine kinase (TyrK) function of Trks. Through its intracellular TyrK domain, TrkB activates the Ras/MAPKinase, PI3Kinase/ AKT and PLCγ signaling pathways downstream [1,2]. Pre-synaptic targets include Synapsin, to trigger vesicle release [5]. Post-synaptic targets include NMDAR and CREB, essential for long-term potentiation, learning and long-term memory [1,5]. Paradoxically, full-length TrkB decays postnatally and TrkB homodimers are not formed in the adult mammalian brain [6–10]. Instead, the most abundant adult isoform is truncated TrkB-T1 lacking the TyrK [8–10]. Mutant mice lacking TrkB-T1 have anxiety, and in humans alterations in TrkB-T1 are linked to severe mental health disorders [11–13]. However, the neuronal functions of the truncated Trk isoforms are poorly understood.

No canonical, bona fide, full-length, TyrK Trk receptors have been found in *Drosophila*. However, neurotrophism, structural and synaptic plasticity, learning and long-term memory all occur in fruit-flies, implying that either in the course of evolution insects and humans found different molecular solutions to elicit equivalent functions, or that undiscovered mechanisms contribute to brain plasticity in both humans and fruit-flies. Finding out what happened to the Trks in *Drosophila* is important, as it could uncover novel fundamental mechanisms of structure-function relationships in any brain.

**Fig 1:**
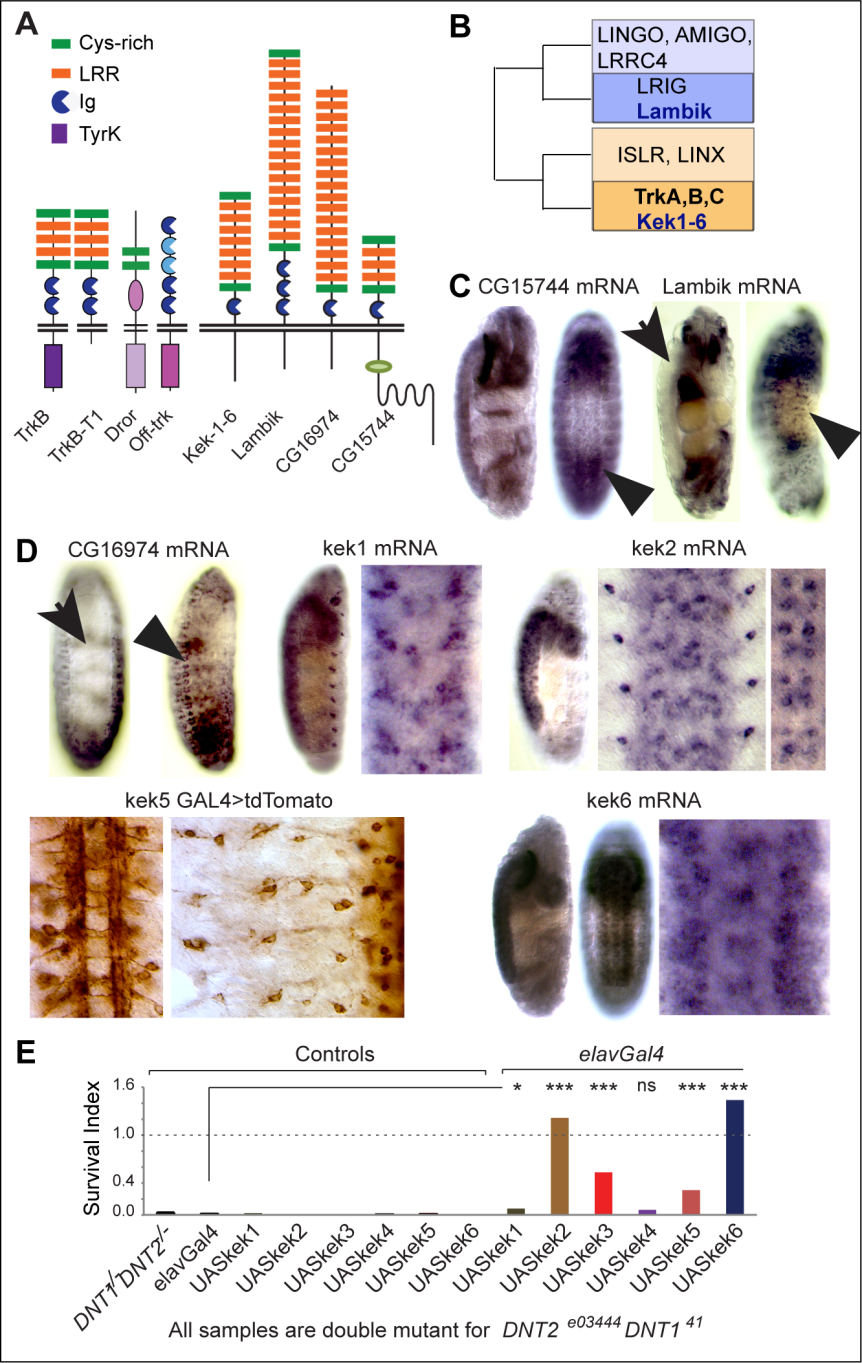
Keks are Trk-like receptors expressed in the CNS. (A) Modular composition of TrkB, TrkB-T1, Dror, Otk and *Drosophila* LIGs. (B) Amongst the LIGs, Keks are closer to the Trks than any other mammalian or *Drosophila* LIGs, adapted from the phylogeny of Mandai et al.[22]. (C,D) mRNA distribution in embryos: *CG15744*, *lambik* and *CG16974* are not expressed in the VNC (arrows) above background, but *lambik* is in PNS and *CG16974* in muscle precursors (arrowheads); *kek-1*, *kek-2* and *kek-6* transcripts are found in the VNC, and *kek5GAL4>tdTomato* drives expression in VNC and PNS (right) neurons. (E) Over-expression of *keks*– most prominently *kek2* and *6* -in all neurons with *elavGAL4* rescued the cold semi-lethality of *DNT1*^*41*^ *DNT2*^*e03444*^ double mutants, n = 52–313 pupae. Chi-square and Bonferroni multiple comparisons correction. *p<0.05, ***p<0.001. For statistical details see S1 Table.

Trk receptors have long been searched for in *Drosophila*. Trks (TrkA,B,C) bear the unique combination of Cysteine Rich Repeats (CRR), Leucine Rich Repeats (LRR) and Immunoglobulin (Ig) domains extracellularly, and an intracellular TyrK domain (Fig 1A). Original searches focused on the TyrK domain, and identified DTrk and Dror as candidate *Drosophila* Trk homologues, but these are unlikely to bind neurotrophins. DTrk, also known as Off-Track (Otk)[14,15] lacks the LRR and CRR modules, it is kinase-dead and binds Semaphorins (Fig 1A). Dror/Dnrk, like all Ror-family receptors, has an extracellular Frizzled/Kringle module instead [16–18](Fig 1A). Subsequent proteomic analyses found no full-length, canonical Trk orthologues with the combination of LRR, CRR and Ig modules extracellularly and a Trk-family TyrK intracellularly in *Drosophila* [19–21]. However, phylogenetic analysis of the Trk-receptor superfamily identified the *Drosophila* Kekkons (Keks), lacking an intracellular TyrK domain, as closely related to the Trks [22](Fig 1A and 1B). Trks and Keks both belong to the LIG family of proteins that contain extracellular ligand-binding LRR and Ig motifs [22,23]. There are 38 LIGs in humans, and amongst these are transmembrane proteins with a divergent intracellular domain lacking a TyrK or any conserved motifs [22]. There are 9 LIGs in *Drosophila*. Phylogenetic analysis clusters mammalian AMIGO, LINGO, LRIG and LRRC4 in one clade with *Drosophila* Lambik, and mammalian Trks in a separate clade together with *Drosophila* Keks (Kek1-6) [22](Fig 1B). Keks are more similar to the Trks than all other vertebrate LIGs are to each other [22]. Keks have only been found in insects, and thus are remnant, conserved Trk-like receptors in fruit-flies.

There are 6 Keks in *Drosophila*, with a cytoplasmic tail lacking any remarkable conservation, except that Kek-1, -2, -5 and -6 have a PDZ domain that could bind cytoplasmic effectors [24]. Kek-1, -2, -5 and -6 are highly conserved in insects [24]. At least *kek-1*, *kek-2* and *kek-5* are expressed in the CNS [25–27]. Only Kek2 has been investigated in the CNS, where it functions as a neuronal activity dependent modulator of synaptic growth and activity [27]. The ligands for the Keks have not been identified.

The prime candidates for Kek ligands are the *Drosophila* neurotrophins (DNTs). DNT1 (*Drosophila* neurotrophin 1 also known as Spz2), DNT2 (also known as Spz5) and Spz bear the distinctive and evolutionarily conserved neurotrophin cystine-knot domain of the mammalian neurotrophins [28–36], and they have conserved CNS functions regulating neuronal survival, connectivity and synaptogenesis [35–39]. Spz, DNT2 and DNT1 are known ligands for Toll-1, Toll-6 and Toll-7 receptors of the Toll and Toll-Like Receptor (TLR) superfamily [38,40]. In *Drosophila* Toll-1, Toll-6 and Toll-7 are required for targeting at the embryonic neuromuscular junction (NMJ), Toll-6 and Toll-8 for larval NMJ growth, and Toll-1, Toll-6 and Toll-7 function as neurotrophin receptors regulating neuronal survival and death, connectivity and behaviour [35–38,41–44]. In *Drosophila*, the NMJ is glutamatergic, and undergoes plasticity and potentiation, thus resembling mammalian central synapses. The NMJ is the standard context in which to investigate synaptic structural and functional plasticity in *Drosophila* [45]. Given that NT family ligands can bind multiple receptor types, and that receptors and ligands tend to co-evolve [17], conservation of the extracellular ligand-binding domain of Trks and Keks suggested Keks could potentially function as DNT receptors in flies.

Here, we investigated whether Kek-6 might function as a DNT receptor in *Drosophila*, at the glutamatergic NMJ synapse.

## Results

### Kek-6 is a truncated-Trk-like receptor that binds DNTs

To investigate if *Drosophila* LIGs might function as DNT receptors in the CNS, we first looked at their expression in embryos. *CG15744* did not reveal expression above background in the ventral nerve cord (VNC) (Fig 1C); *lambik* and *CG16974* mRNAs were absent from the VNC, but might be expressed in the Peripheral Nervous System (PNS) or muscle, respectively (Fig 1C and 1D). *kek1* and *kek2* were expressed in VNC cells, and possibly in PNS cells too (Fig 1D), as previously shown [25,27]. *kek-3* and *kek-4* transcripts were not detected in embryonic CNS. *kek-5* expressed in the VNC [26], and we confirmed this with a *kek5-GAL4* reporter, which also revealed PNS expression (Fig 1D). *kek-6* transcripts were abundant in the VNC (Fig 1D). Thus, amongst the 9 LIGs, Kek-1, -2, -5, and -6 could function in the CNS.

To test whether Keks could function downstream of DNTs in vivo, we took advantage of the cold semi-lethality of *DNT1*^*41*^ *DNT2*^*e03444*^ double mutants [38], and asked whether it could be rescued with the over-expression of *keks* in neurons. Over-expression of *kek-1* and *kek-4* in neurons (with *elavGAL4)* did not rescue, and *kek-2* and *kek-6* did most prominently (Fig 1E, S1 Table). Thus, Kek-2 and Kek-6 could function downstream of DNTs.

**Fig 2:**
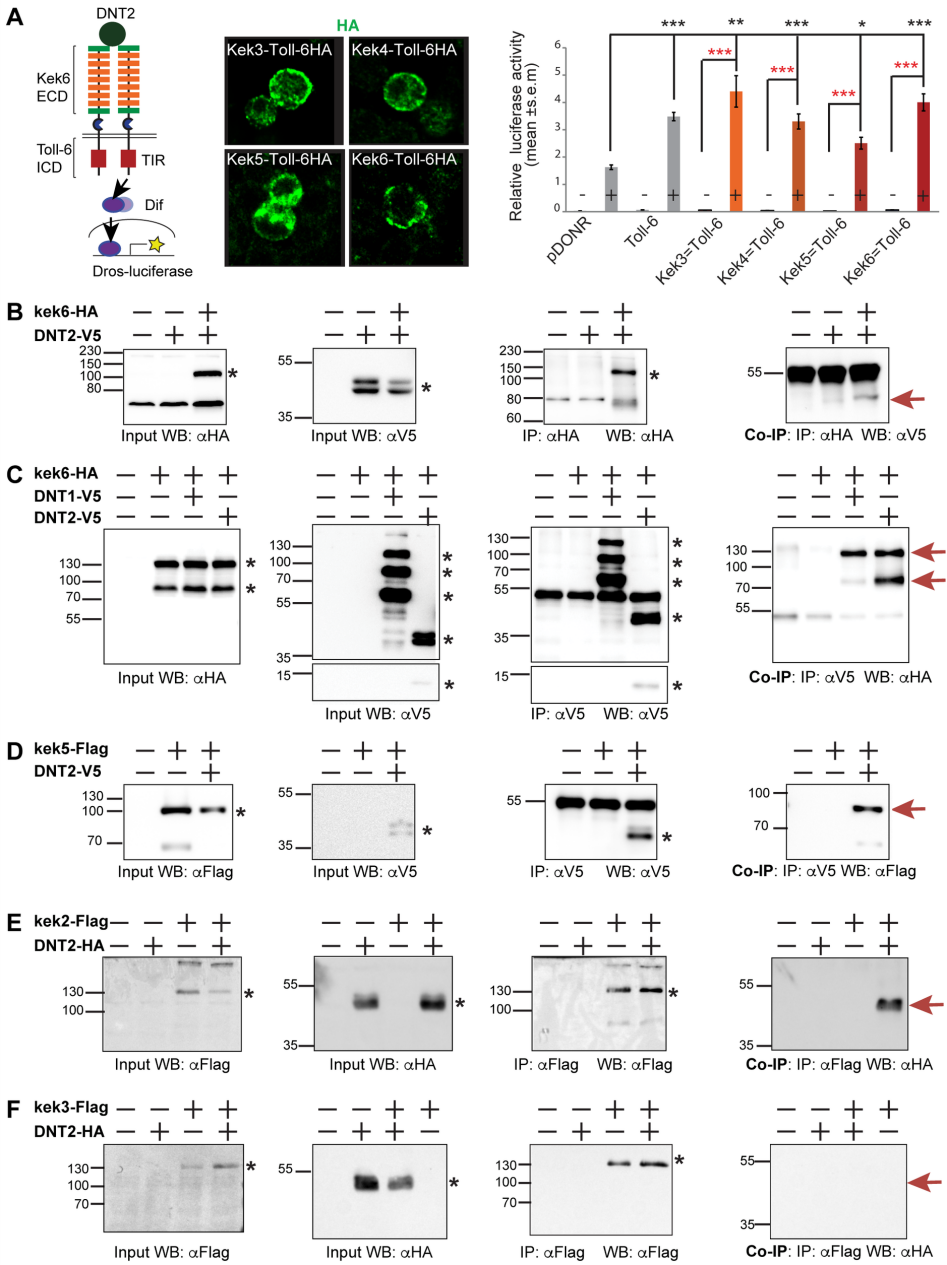
Keks bind DNTs. (A) Diagram of Chimaeric Kek-Toll-6-HA receptors, bearing the extracellular and transmemebrante domains of Keks and the intracellular signaling domain of Toll-6 (left). Chimaeric receptors visualized with anti-HA antibodies are distributed to the cell membrane in S2 cells (middle). Binding of purified mature DNT2-CK to the extracellular domain of Kek-Toll-6 receptors activates the *drosomycin-luciferase* reporter (right). Black asterisks denote comparisons to empty vector (pDONR) control, and red asterisks refer to no-stimulation controls. Welch ANOVA p<0.001; asterisks refer to post-hoc Games-Howell *p<0.05, **p<0.01, ***p<0.001. n = 9 (3x in triplicate). (B-F) Western blots showing co-immunoprecipitations from co-transfected S2 cells: (B) precipitation of Kek6-HA with anti-HA brings down DNT2-V5 detected with anti-V5. (C) Reverse co-IP for Kek6 and DNT2. Precipitation of DNT1-V5 also brings down Kek6-HA. (D) Precipitation of DNT2-V5 brings down Kek5-Flag. (E) Precipitation of Kek2-Flag brings down DNT2-HA. (F) Precipitation of Kek3-Flag failed to bring down DNT2-HA. Black asterisks indicate the relevant control bands, red arrows indicate co-IPs. Input controls: cell lysate from co-transfected cells express both proteins. IP: immunoprecipitation; WB: western blot. DNTs can be spontaneously cleaved following expression from these constructs in S2 cells.

To ask whether DNT ligands could bind Keks and induce signaling, as Keks lack the TyrK, to enable a signaling readout, we generated chimaeric receptors formed of the extracellular and transmembrane domains of the Keks fused to the intracellular domain of Toll-6 (Fig 2A). Toll-6 uses a conserved TIR domain to activate Dif/NFκB signaling downstream, which can be measured through the activation of *drosomicin-luciferase (dros-luc)* [38]. We used S2 cells stably transfected with *dros-luc*, transfected them with *kek-Toll-6* chimaeric receptors, and tested whether stimulation with purified cleaved DNT2 (DNT2-CK) could induce Dif/NFκB signaling. The chimaeric receptors targeted correctly to the S2 cell membrane (Fig 2A). Stimulation with DNT2-CK in *pDONR* transfected controls induced *dros-luc*, as S2 cells express multiple Toll receptors [36,38] (Fig 2A). Stimulation with DNT2-CK of cells transfected with *kek3*,*4*,*5*,*6-Toll-6* chimaeras had an effect comparable to the induction by stimulated full-length *Toll-6*, and Kek3-Toll-6 and Kek6-Toll-6 chimaeras responded more robustly (Fig 2A, S1 Table). We could not generate Kek-1 and Kek-2 chimeric receptors, thus a potential interaction with them cannot be ruled out. Thus, DNT2 can interact physically with at least Kek-3 and Kek-6.

To verify whether Kek-6 could bind DNT2, we carried out co-immunoprecipitations. S2 cells were co-transfected with HA-tagged *kek-6* and V5-tagged full-length *DNT2*. Precipitating Kek6-HA with anti-HA, brought down DNT2-V5 detected with anti-V5 (Fig 2B). Conversely, precipitating DNT2 with anti-V5, also brought down Kek6 detected with anti-HA (Fig 2C). Thus, Kek6 can bind DNT2. To test whether Kek-6 might also bind DNT1, we co-transfected S2 cells with *kek6-HA* and full-length *DNT1-V5*. Precipitating DNT1 brought down Kek-6 (Fig 2C). Thus, Kek-6 can bind both DNT2 and DNT1. To test if DNT2 could bind other Keks, S2 cells were co-transfected with *kek5-Flag* and *DNT2-V5*, and precipitating DNT2 also brought down Kek-5 (Fig 2D). Similarly, S2 cells were co-transfected with *kek2-Flag* and *DNT2-HA*, and precipitating Kek-2 also brought down DNT2 (Fig 2E). However, upon co-transfection, precipitating Kek3-Flag failed to robustly bring down DNT2-HA (Fig 2F). Thus, DNT2 can equally bind Kek-5 and Kek-2, but cannot bind Kek-3 as well. These data demonstrate that DNT ligands bind Keks, that binding is somewhat promiscuous, and that DNTs might preferentially bind the CNS-specific Kek-2, -5 and -6.

As Kek-6 was widely expressed in the CNS, rescued the semi-lethality of *DNT1 DNT2* double mutants, and bound DNT ligands in both a signaling assay and in co-immunoprecipitations, from this point we focused on Kek-6. We asked whether Kek-6 could function as a DNT receptor.

### Kek-6 in motoneurons and DNT2 from the muscle function together at the NMJ

**Fig 3:**
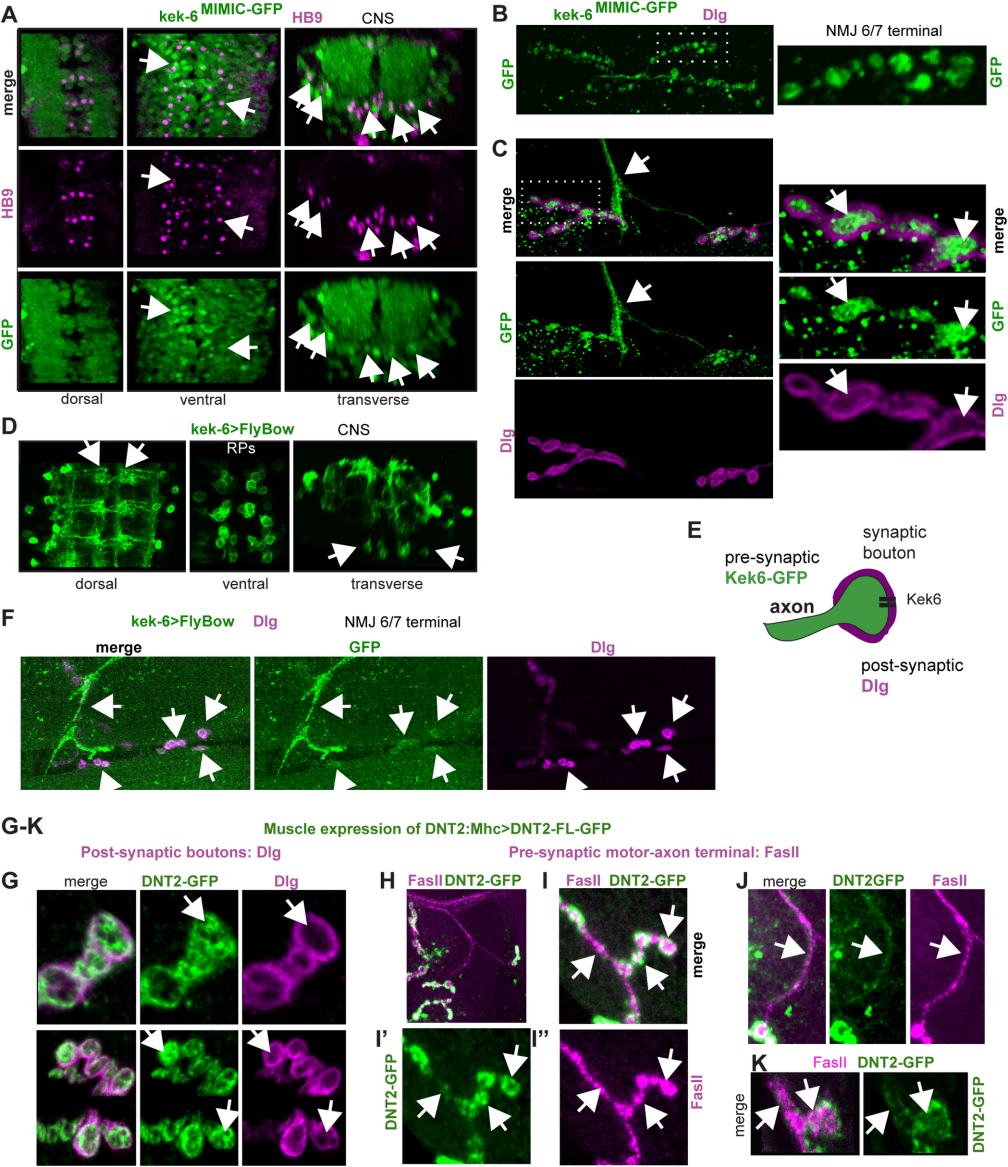
Kek-6 is expressed pre-synaptically in motoneurons and binds post-synaptic DNT2. (A) In Kek-6^GFP^ larval VNCs, GFP colocalises with the neuronal marker HB9 (arrows show examples). (B) Kek-6^GFP^ was found in third instar larval muscle 6/7 NMJ and synaptic boutons (dotted rectangle: higher magnification, right). (C) Kek-6^GFP^ was found in the motoneuron axonal terminal (arrows), and in pre-synaptic bouton lumen (dotted rectangle: higher magnification, right), not colocalising with the post-synaptic marker anti-Dlg (arrows).(D) Kek-6>FlyBow was localized to CNS axons and dendrites (arrows), and cell bodies of the RP3,4,5 motoneuron clusters (ventral and transverse views, arrows). (E) Illustration. (F) Kek-6>FlyBow was also distributed along the motoneuron axons, NMJ terminal (arrow) and synaptic boutons (arrows). (G-K) Over-expression of GFP tagged full-length DNT2 in muscle *(MhcGAL4>UAS-DNT2-FL-GFP)* revealed: (G) DNT2-GFP distribution within the pre-synaptic bouton lumen (arrows), boutons labeled post-synaptically with anti-Dlg; (H-K) DNT2-GFP along the motoraxon (labeled with anti-FasII) and within the pre-synaptic bouton lumen (arrows).

For a functional *in vivo* analysis we focused on Kek-6. *kek-6* CNS expression was examined in larvae using *kek6*^*MIMIC13953*^ flies bearing a GFP insertion into the *kek-6* coding region (hereafter named Kek6^GFP^). Kek6^GFP^ was found in HB9+(Fig 3A) and Eve+(S1A Fig) inter-neurons and motoneurons, and excluded from Repo+ glia (S1B Fig) in third instar larval VNCs. Kek6^GFP^ was present in motoneuron terminals at the neuro muscular junction (NMJ) of third instar larvae and not in the muscle (Fig 3B and 3C), revealing NMJ6/7 synaptic boutons, surrounded by post-synaptic Dlg (i.e. *Drosophila* PSD95) (Fig 3C). To verify this, we swapped *MIMIC* for *GAL4* using RMCE, and drove expression of the membrane tethered *FlyBow* reporter. *kek6>FlyBow* revealed abundant signal in CNS neurons, axons and dendrites, and in the cell body clusters of RP3,4,5 motoneurons (Fig 3D). *kek6>FlyBow* was also present in motoraxons reaching the NMJ, and in synaptic boutons (Fig 3F). No prominent signal was detected in the muscle above background levels. However, data do not exclude potential post-synaptic distribution in the CNS. To conclude, *kek-6* is expressed pre-synaptically in motoneurons (Fig 3E).

*DNT2* transcripts are expressed in the larval body wall muscles, and localised to post-synaptic boutons [39]. To test if DNT2 could function retrogradely, we generated a tagged form of full-length DNT2 with GFP at the C-terminus, and over-expressed *DNT2-FL-GFP* in the muscle with *MhcGAL4*. DNT2-FL-GFP would result in secretion of mature DNT2-CK-GFP [36], although not all protein might get cleaved and secreted. Over-expression of *DNT2-FL-GFP* in muscle resulted in the localization of GFP pre-synaptically in boutons, surrounded by the post-synaptic marker Dlg (Fig 3G). DNT2-FL-GFP also colocalised with the motoneuron marker FasII in boutons (Fig 3H, 3I and 3K) and motoraxons (Fig 3I, 3J and 3K). Thus, DNT2 produced in muscle could get distributed to the motoneuron, consistent with a retrograde function.

**Fig 4:**
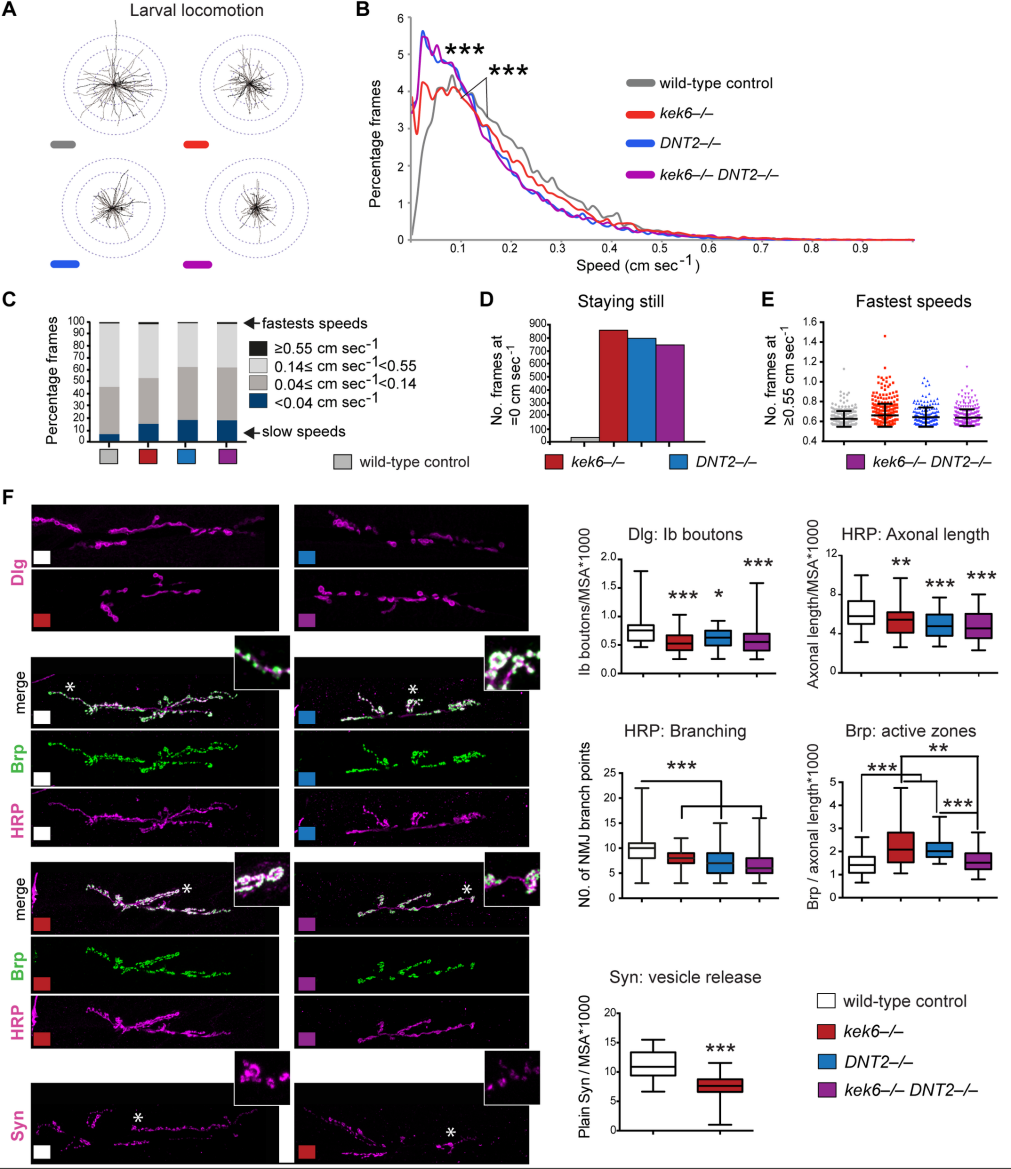
*kek6* and *DNT2* mutants have smaller NMJs and impaired locomotion. (A) Plotted trajectories of filmed larvae, and (B) histograms of percentage frames at each speed analysed with FlyTracker. Kruskal-Wallis p<0.0001 and ***p<0.001 post-hoc Dunn test, n≥22344 frames. (C-E) Speed distribution classified into arbitrary categories. (C) Mutants spend more time at the lowest speeds than controls, generally do not crawl at the higher speeds (pale grey, left), but like controls can reach the highest speeds for a small fraction of time. (D) Wild-type larvae are hardly at speed = 0, contrary to the mutants. (E) All genotypes can achieve the highest speeds, but none spend much time crawling at these speeds. (F) NMJs (left, with higher magnification details of areas indicated by asterisks) and box-plot graphs (right) showing: *kek-6*^*–/–*^and *DNT2*^*–/–*^single mutants and *kek-6*^*–/–*^*DNT2*^*-/-*^double mutants have fewer Dlg+ boutons, smaller HRP+ axonal terminals (normalized to muscle area, MSA), and less complex NMJs with reduced axonal branching. Dlg: Kruskal-Wallis p<0.0001, and *p<0.05, **p<0.01, ***p<0.001 post-hoc Dunn; HRP: One Way ANOVA p<0.0001, and **p<0.01, ***p<0.001 post-hoc Dunnett. *kek-6* and *DNT2* single mutants, but not the double mutants, have increased active zone density (Brp+/HRP+axonal length). Brp: Kruskal-Wallis p = 0.0012, and **p<0.01, ***p<0.001 Dunn’s post-hoc. *Kek-6* mutants have reduced Synapsin, Mann-Whitney U test ***p<0.001. For statistical details, see S1 Table. N = 30–113 hemisegments. Mutant genotypes throughout figures: Control: *yw/+;* Mutants: *kek-6*^*–/–*^: *kek6*^*34*^*/Df(3R)6361; DNT2*^*–/–*^: *DNT2*^*37*^*/Df(3L)6092; kek-6*^*–/–*^*DNT2*^*–/–*^: *kek6*^*34*^*Df(3L)6092/* Df(3R)6361 *DNT2*^*37*^.

To investigate the *in vivo* functions of Kek-6 and DNT2, we generated *kek-6* and *DNT2* null mutant alleles by FRT-mediated recombination between PiggyBac insertions [46] (S2 Fig). Neither *kek6*^*34*^*/Df(3R)ED6361* or *DNT2*^*37*^*/Df(3L)6092* loss of function mutants, nor *kek6*^*–/–*^*DNT2*^*–/–*^double mutants, affected viability. We analysed larval locomotion using FlyTracker software to trace trajectories and measure crawling speed [38], and found that *kek6*^*34*^*/Df(3R)ED6361* mutant larvae crawled more slowly than controls (Fig 4A and 4B, S1 Table), and *DNT2*^*37*^*/Df(3L)6092* larvae crawled even slower (Fig 4A and 4B, S1 Table). *kek6*^*–/–*^*DNT2*^*–/–*^double null larvae crawled at similar speeds as *DNT2*^*–/–*^mutants, but travelled shorter distances, moving around the starting spot (Fig 4A and 4B, S1 Table). Overall, *DNT2*^*–/–*^mutants and the double mutants crawled slowest (Fig 4C). Furthermore, *kek6*^*–/–*^*and DNT2*^*–/–*^single mutants and *kek6*^*–/–*^*DNT2*^*–/–*^double mutants spent considerably longer times than controls not moving (Fig 4D). However, when crawling, both single mutants, and *kek6*^*–/–*^*DNT2*^*–/–*^double mutants could attain the fastest speeds achieved by controls (Fig 4C and 4E). All genotypes only spent very brief times at these high speeds (Fig 4C and 4E). The shared phenotypes of single and the double mutants, and synergistic effect in the doubles (Fig 4A), suggested that Kek-6 and DNT2 might be functionally linked.

Locomotion phenotypes suggested the NMJ might be affected, so we looked at the muscle 6/7 NMJs, which require DNT1 and 2 [39]. Targeting at the embryonic muscle 6/7 NMJ was affected in *kek6*^*–/–*^mutants and upon *kek-6* over-expression in neurons (S3 Fig). In wandering third instar larvae, *kek6*^*–/–*^and *DNT2*^*–/–*^single mutant larvae, and *kek6*^*–/–*^*DNT2*^*–/–*^double mutants, had smaller NMJs than controls, with fewer Ib boutons and shorter axonal terminal length (Fig 4F, S1 Table). All mutant genotypes also had reduced NMJ branching (Fig 4F). Thus, Kek-6 and DNT2 are required for normal NMJ growth. *kek6*^–/–^and *DNT2*^*–/–*^single mutant NMJs had higher active zone density, visualized with anti-Brp (*Drosophila* ELKs) and quantified automatically throughout the NMJ stack of images using DeadEasy Synapse software [39](Fig 4F, S1 Table). Since the NMJs were smaller, this suggested that the increase in active zones was a homeostatic compensation of defective synaptic function, to enable adequate behaviour. Homeostatic adjustments in active zones are a common manifestation of structural plasticity at the NMJ [47]. Remarkably, the increase in active zone density did not occur in *kek-6*^*–/–*^*DNT2*^*–/–*^double mutants (Fig 4F, S1 Table), meaning that compensation fails in the double mutants. To further test how Kek-6 affects the synapse, we also visualised Synapsin, which phosphorylates components of the SNARE complex to promote neurotransmitter vesicle release [48], and quantified it automatically using DeadEasy Synapse software [39]. *kek6*^–/–^mutants had reduced Synapsin production at synaptic locations (Fig 4F, S1 Table), revealing defective synapse composition. These data show that: (1) Kek-6 is required for appropriate synaptic structure; (2) that in *kek-6*^*–/–*^mutants homeostatic compensation regulates Brp but not Synapsin; (3) and that DNT2 is involved in the compensation mechanism, as it fails in its absence.

**Fig 5:**
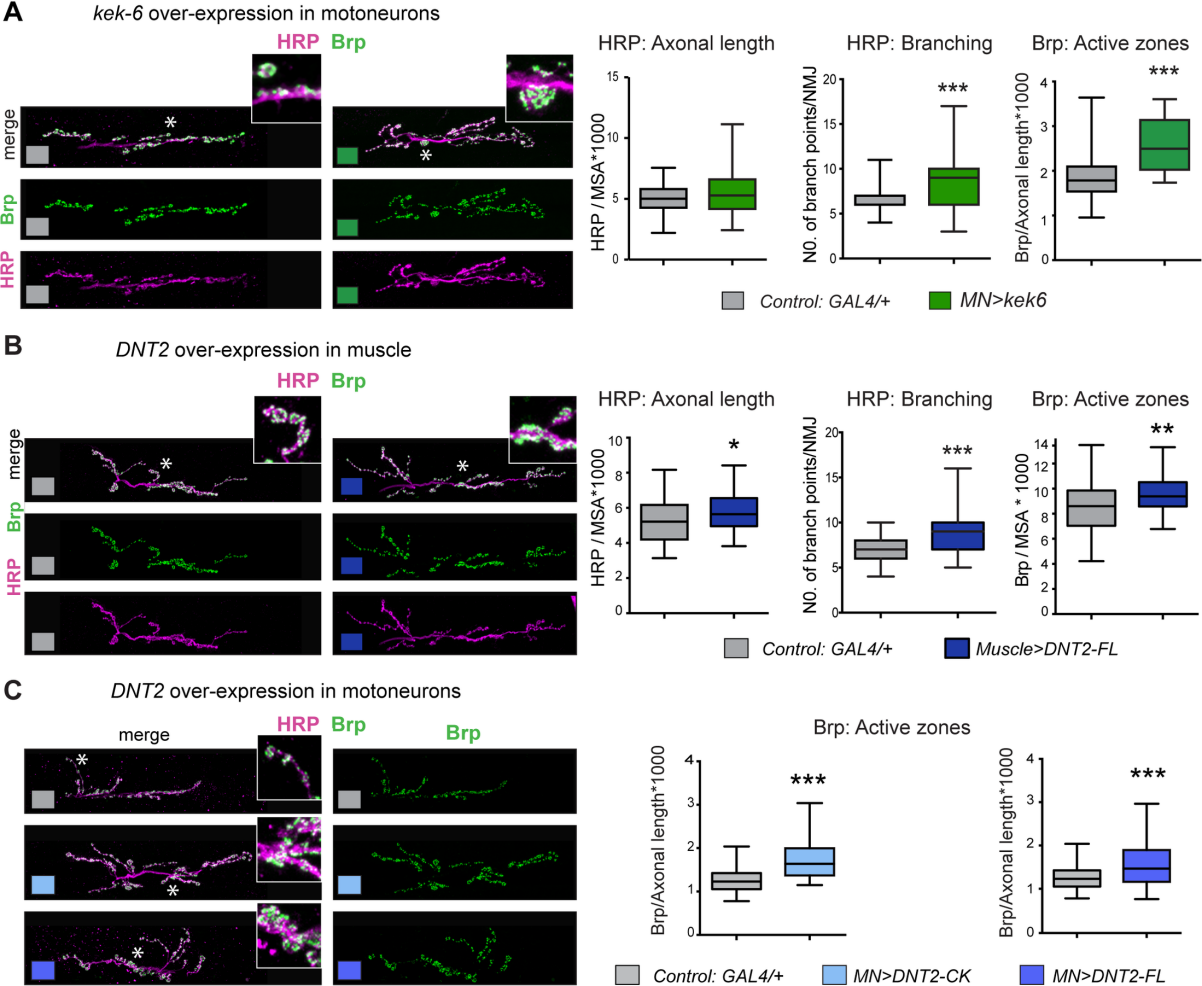
Kek-6 and DNT2 can induce active zones and NMJ growth. Confocal images of NMJs from A3-4 muscle 6/7 (left, higher magnification deail of areas indicated by asterisks), and box-plot graphs (right), showing: (A) Over-expression of *kek6* in motoneurons had no effect on HRP+ NMJ size, but it increased Brp+ active zones. HRP: Student t test n.s. p = 0.07; Brp: Mann-Whitney U test ***p<0.001.(B) Over-expression of full-length DNT2 in muscle increased NMJ size (HRP) and active zones (Brp), revealing a retrograde function. HRP: Mann-Whitney U test *p<0.05; Brp: Student t test **p<0.01. (C) Over-expression of both full-length DNT2 and mature DNT2-CK in motoneurons induced active zone formation. Brp DNT2-CK: Student t test **p<0.01, and Brp DNT2-FL: Mann-Whitney U test ***p<0.001. n = 29–66 hemisegments. See S1 Table. *MN = D42GAL4; Muscle = MhcGAL4*.

**Fig 6:**
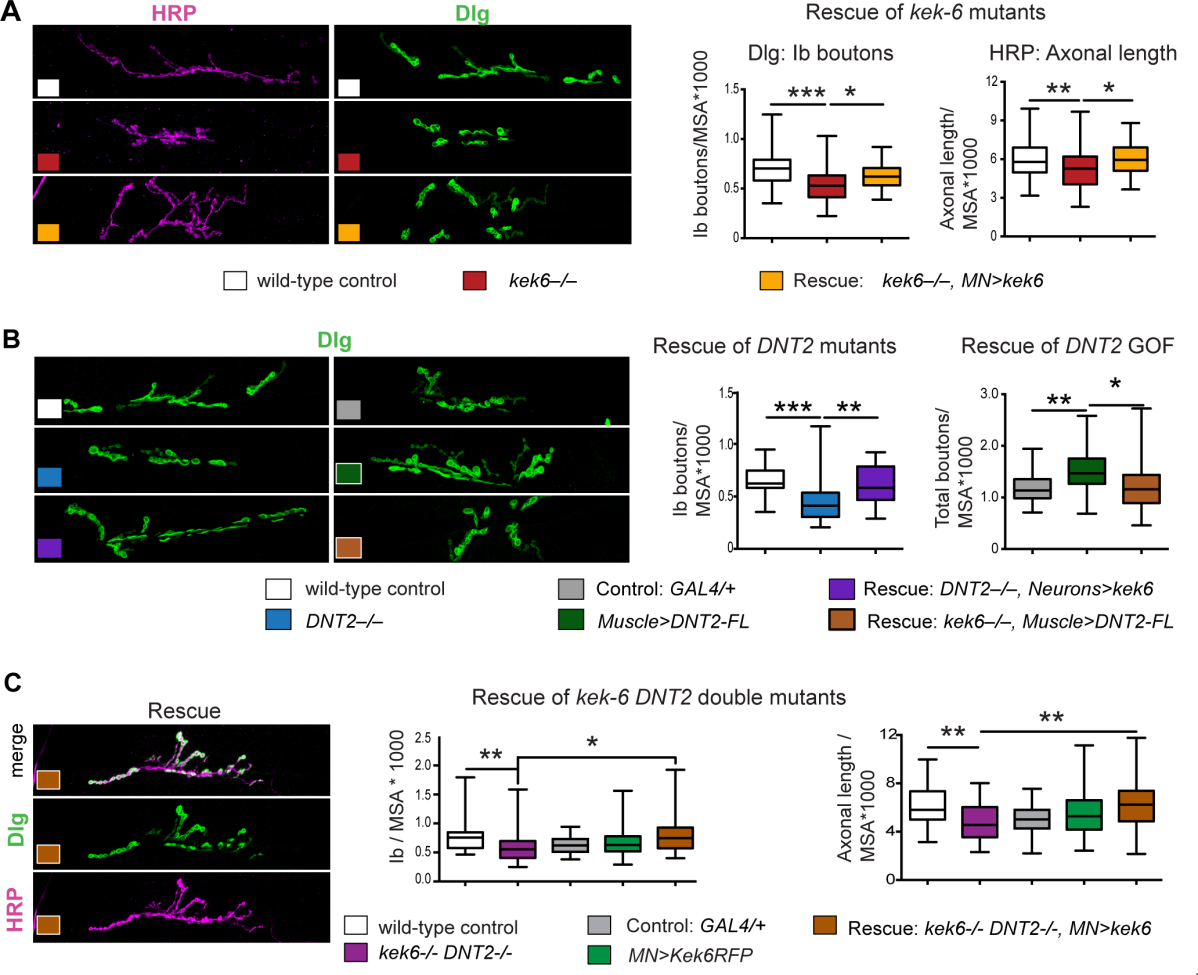
Kek-6 functions downstream of DNT2. Confocal images of NMJs from A3-4 muscle 6/7 (left), and box-plot graphs (right), showing: (A) Over-expression of *kek-6* in motoneurons rescued the phenotypes of *kek-6* mutants. Dlg: One Way ANOVA p<0.0001 and post-hoc Bonferroni *p<0.05, ***p<0.001. HRP: One Way ANOVA p<0.001 and post-hoc Bonferroni **p<0.01, **p<0.01. (B) Left: Over-expression of *kek-6* in neurons rescued the phenotype of *DNT2* mutants. Dlg: Kruskal-Wallis p = 0.001, and post-hoc Dunn test **p<0.01, ***p<0.001. Right: *kek-6* loss of function rescued the increase in boutons caused by the muscle over-expression of *DNT2*. Dlg: Welch ANOVA p<0.01 and post-hoc Games-Howell *p<0.05, **p<0.01. (C) Over-expression of *kek-6* in motoneurons rescued the phenotypes of *kek-6 DNT-2* double mutants. Dlg: Kruskal-Wallis p = 0.001 and post-hoc Dunn’s test *p<0.05, **p<0.01. HRP: Welch ANOVA p = 0.000, post-hoc Games Howell **p<0.01. n = 29–101 hemisegments. See S1 Table. GAL4 drivers: Muscle: *MhcGAL4*; Neurons: *elavGAL4;* MN: *D42 or Toll-7GAL4*. Controls: white boxes: yw/+; grey boxes: GAL4/+; mutant genotypes as in Fig 4. Rescue genotypes: (A) *w; UASkek6RFP/+; Df(3R)6361/kek6*^*34*^*D42GAL4*. (B) *w; UASkek6RFP/+; elavGAL4 Df(3L)6092/ DNT2*^*37*^; and *w; UASDNT2-FL/+; Df(3R)6361/kek6*^*34*^*D42GAL4*. (C) *w; Toll-7GAL4/UASkek6RFP; kek6*^*34*^*Df(3L)6092/ Df(3R)6361 DNT2*^*37*^.

Over-expression of *kek-6* in motoneurons alone did not alter NMJ size, but increased branching (Fig 5A and S4A and S4B Fig) and active zone density (Fig 5A, S1 Table). It also induced ghost boutons, albeit not significantly (similarly in *kek-6*^*–/–*^mutants, S4C–S4E Fig, S1 Table). Ghost boutons are presynaptic, HRP+ Dlg-negative, immature boutons that fail to get stabilized, and are correlates of increased neuronal activity. Together, these data are consistent with Kek-6 influencing synaptic structure and/or function. Over-expression of *DNT2-FL* in muscle increased axonal terminal size and branching (Figs 5B and 6B). Furthermore, over-expression of full-length *DNT2-FL* in muscle, and either full-length *DNT2-FL* or mature *DNT2-CK* in motoneurons (with *D42GAL4*), also increased active zones (Fig 5B and 5C, S1 Table). Thus, DNT2 can affect NMJ size and synaptic structure.

Altogether, these data showed that both Kek-6 and DNT2 are required: (1) for NMJ growth, (2) for appropriate synaptic structure. Furthermore, whereas *kek-6* cannot promote NMJ growth, DNT2 can.

Data suggested that DNT2 may function as a retrograde ligand for Kek-6. To further test this, we used epistasis analysis. Over-expression of *kek6* in motoneurons rescued the NMJ mutant phenotypes of bouton number and axonal length of *kek6*^*34*^*/Df(3R)6361* mutants (Fig 6A, S1 Table), demonstrating that the *kek6*^*–/–*^mutant phenotypes were specific. Over-expression of *kek6* in motoneurons rescued the NMJ phenotypes of *DNT2*^*37*^*/Df(3L)6092* mutants and *kek6*^*–/–*^*DNT2*^*–/–*^double mutants (Fig 6B and 6C, S1 Table), demonstrating that Kek-6 functions downstream of DNT2. Over-expression of untagged *DNT2-FL* in muscle (with *MhcGAL4*) increased Dlg+ bouton number (Fig 6B), and importantly, this was rescued by *kek-6* loss of function (Fig 6B, S1 Table). Since Kek-6 is expressed in motoneurons and functions pre-synaptically, and DNT2 was over-expressed in muscle, this demonstrates that DNT2 is a retrograde ligand for Kek-6. Altogether, our data demonstrate that DNT2 is a retrograde ligand for Kek-6 at the NMJ and that DNT2 and Kek-6 are required together for synaptic structure and NMJ growth.

Intriguingly, our data also suggested that DNT2 had additional functions compared to *kek-6*. Firstly, the homeostatic compensation of active zones seen in *kek6*^*–/–*^mutants did not occur in *kek-6*^*–/–*^*DNT2*^*–/–*^double mutants (Fig 4F). This suggests that homeostatic compensation depends on an alternative mechanism downstream of DNT2. Secondly, over-expression of *DNT2* but not *kek-6* increased NMJ size, as both HRP+ axonal terminal length (Fig 5B) and bouton number (Fig 6B) increased when *DNT2-FL* was over-expressed from muscle. This suggests that a second mechanism downstream of DNT2 can influence NMJ growth. DNT2 is a known ligand of Toll-6 [38], and Toll-6 and -8 are required in motoneurons for NMJ growth and active zone formation[37,43]. So this raised two questions: do Toll-6 and Kek-6 interact functionally as DNT2 receptors at the NMJ, and why?

### Kek-6 and Toll-6 cooperate to modulate NMJ structural plasticity

LIG proteins and truncated Trk isoforms can function as ligand sinks or dominant negative co-receptors that abrogate signaling, e.g. by full-length Trks [6,7]. Thus, Kek-6 might inhibit Toll-6 function.

**Fig 7:**
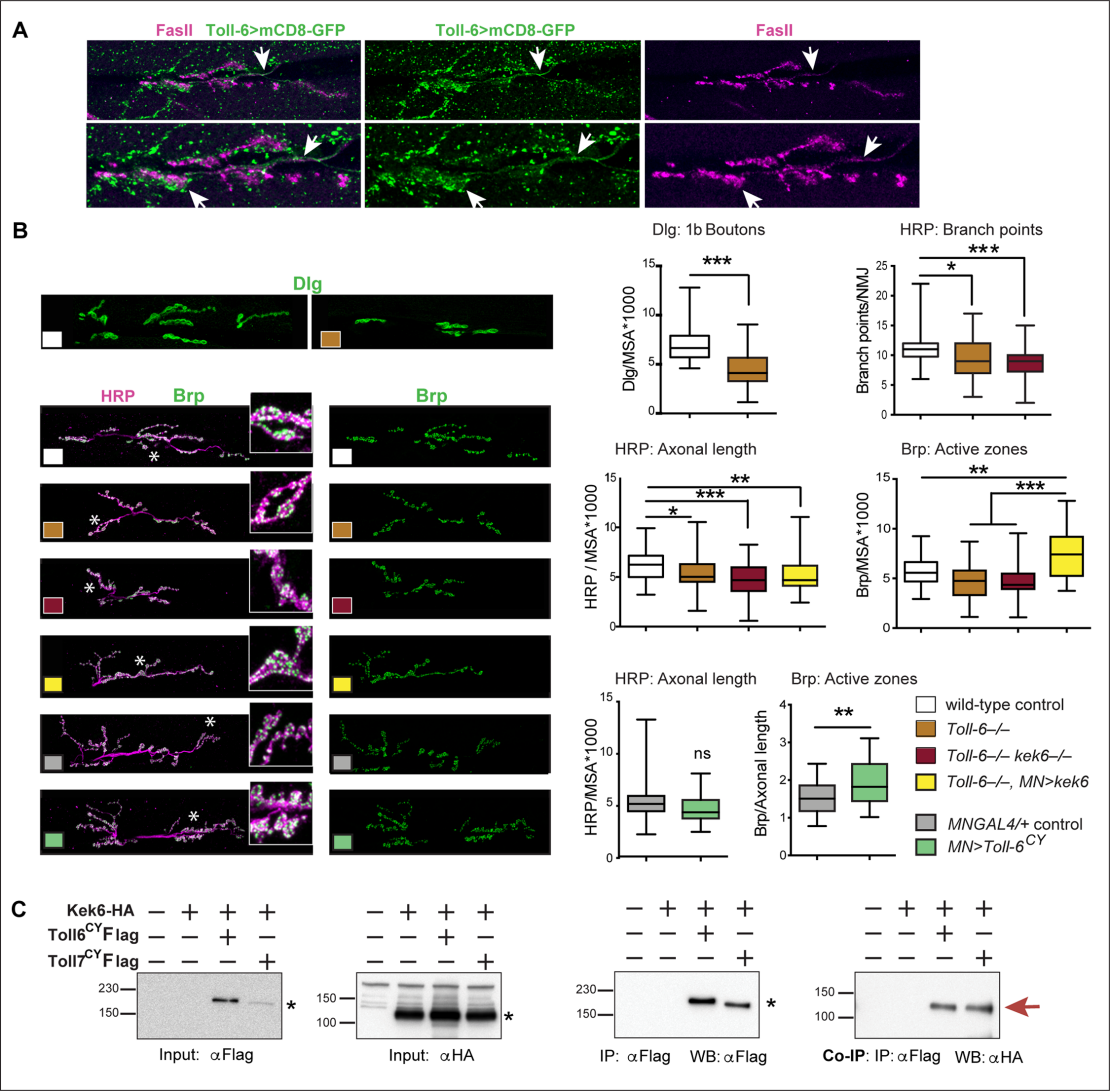
Kek-6 and Toll-6 interact for NMJ structural homeostasis. (A) Toll-6GAL4>mCD8-GFP is distributed in FasII+ motoneuron axons (arrows) at the muscle 6/7 NMJ terminal. (B) Muscle 6/7 NMJs (left) and box-plot graphs (right) showing: *Toll-6*^*MIO2127*^*/Df(3L)BSC578* mutants had fewer 1b boutons. *Toll-6*^*–/–*^and *Toll-6*^*MIO2127*^*Df(3R)6361/kek6*^*35*^ *Df(3L)BSC578* double mutants had smaller NMJs (HRP, Kruskal-Wallis p = 0.0001) with reduced branching, and reduced active zones (Brp, Kruskal-Wallis p = 0.0055), post-hoc Dunn for both *p<0.05, ***p<0.001. Pre-synaptic over-expression of *kek-6* in motoneurons in Toll-6^-/-^mutants (*w; UASkek-6/+; Toll-6*^*MIO2127*^*GAL4/ Df(3L)BSC578*) did not rescue NMJ size, but upregulated Brp+. Over-expression of activated *Toll-6*^*CY*^ did not affect NMJ size (HRP) but increased active zones. N = 34–46 hemisegments. (C) Co-immunoprecitation from co-transfected S2 cells: Precipitating Toll-6 and Toll-7 with anti-Flag brought down Kek-6 detected with anti-HA. IP: immuno-precipitation; WB: western blot; asterisk: co-IP. See S1 Table. *MN = D42GAL4*.

Toll-6 was shown to function at the larval NMJ [43], but its expression here had not been reported. Using a *MIMIC* insertion into the intronless coding region of Toll-6, we generated *Toll-6GAL4* flies by RMCE. Toll-6>mCD8-GFP was distributed along the motoraxon of the muscle 6/7 NMJ (Fig 7A). Like *kek-6*^*–/–*^and *DNT2*^*–/–*^mutants, *Toll-6*^*MIO2127*^*/ Df(3L)BSC578* mutants had smaller NMJs, with fewer 1b boutons (Fig 7B), shorter HRP+ axonal length (Fig 7B, S1 Table) and reduced branching (Fig 7B) than controls. This confirms that Toll-6 is required for NMJ growth. Contrary to *kek-6*^*–/–*^mutants, *Toll-6*^*–/–*^mutants had reduced Brp+ active zones compared to controls (Fig 7B), meaning that Toll-6 is required for active zone formation. *kek-6*^–/–^*Toll-6*^*–/–*^double mutant larvae also had small NMJs (Fig 7B, S1 Table), with reduced branching complexity (Fig 7B), both consistently more pronounced than the single *Toll-6* mutants when compared to controls. Importantly, Brp+ active zones remained lower than in controls and comparable to the levels of *Toll-6*^*–/–*^single mutants (Fig 7B). Thus, the compensatory increase in active zones observed in *kek-6*^*–/–*^single mutants did not occur in the absence of *Toll-6*. Conversely, over-expression of *Toll-6* did not alter NMJ size, but increased active zones (Fig 7B). Thus, Toll-6 can induce Brp+ active zone formation. Together, these data show that active zone formation and homeostasis depend on Toll-6.

Evidence indicated that Brp depends on Toll-6 and not Kek-6, but both Kek-6 and Toll-6 could induce Brp+ active zones. This could mean that Kek-6 might induce Brp+ indirectly via Toll-6. To test this, we asked whether Kek-6 could induce active zone formation in the absence of Toll-6. Over-expression of *kek-6* presynaptically in motoneurons in *Toll-6*^*–/–*^mutants increased Brp+ (Fig 7B, yellow). This demonstrates that Kek-6 can induce active zone formation independently of Toll-6.

These results revealed that: 1) Loss of both *kek-6*^*–/–*^and *Toll-6*^*–/–*^more robustly reduced NMJ size and complexity than loss of *Toll-6*^*–/–*^alone, suggesting that Kek-6 contributes to NMJ growth via an alternative mechanism independently of Toll-6. 2) NMJ growth requires DNT2, Kek-6 and Toll-6. However, over-expression of *DNT2* had a much stronger effect than over-expressing either *kek-6* or *Toll-6* alone. This means that NMJ growth is regulated by the concerted functions of both DNT2 receptors, Toll-6 and Kek-6. 3) Synaptic structure requires Toll-6 and Kek-6, but via distinct parallel mechanisms. 4) The compensatory increase in active zones observed in *kek-6*^*–/–*^and *DNT2*^*–/–*^single mutants did not occur in *kek-6*^*–/–*^*DNT2*^*–/–*^or *kek-6*^*–/–*^*Toll-6*^*–/–*^double mutants, meaning that the concerted functions of Toll-6 and Kek-6 are required for NMJ structural synaptic homeostasis. 5) Since Toll-6 is required for active zone formation and NMJ growth, and over-expression of *kek-6* increased active zones and did not compromise NMJ growth, Kek-6 does not function as a ligand sink, an inhibitor or dominant negative co-receptor to abrogate Toll-6 function.

The data suggested that Kek-6 and Toll-6 can function together forming a receptor complex for DNT2. To test whether Kek-6 and Toll-6 might physically interact, we carried out co-immunoprecipitations. S2 cells were co-transfected with the active forms *Toll-6*^*CY*^*-Flag* and *Kek6-HA*, and we found that precipitating Toll-6^CY^ with anti-Flag brought down Kek6 detected with anti-HA (Fig 7C). Similarly, precipitating Toll-7^CY^ also brought down Kek6 (Fig 7C). Thus, Kek6 can bind Toll-6, and also Toll-7.

Altogether, these data showed that retrograde DNT2 can bind Toll-6 and Kek-6, which can interact pre-synaptically to form a receptor complex. They cooperatively promote NMJ growth and regulate synaptic structure. This raised a new question: if Kek-6 could influence NMJ growth and synaptic structure independently of Toll-6, and without a TyrK, how might it function?

### Kek-6 functions via CaMKII and VAP33A

**Fig 8:**
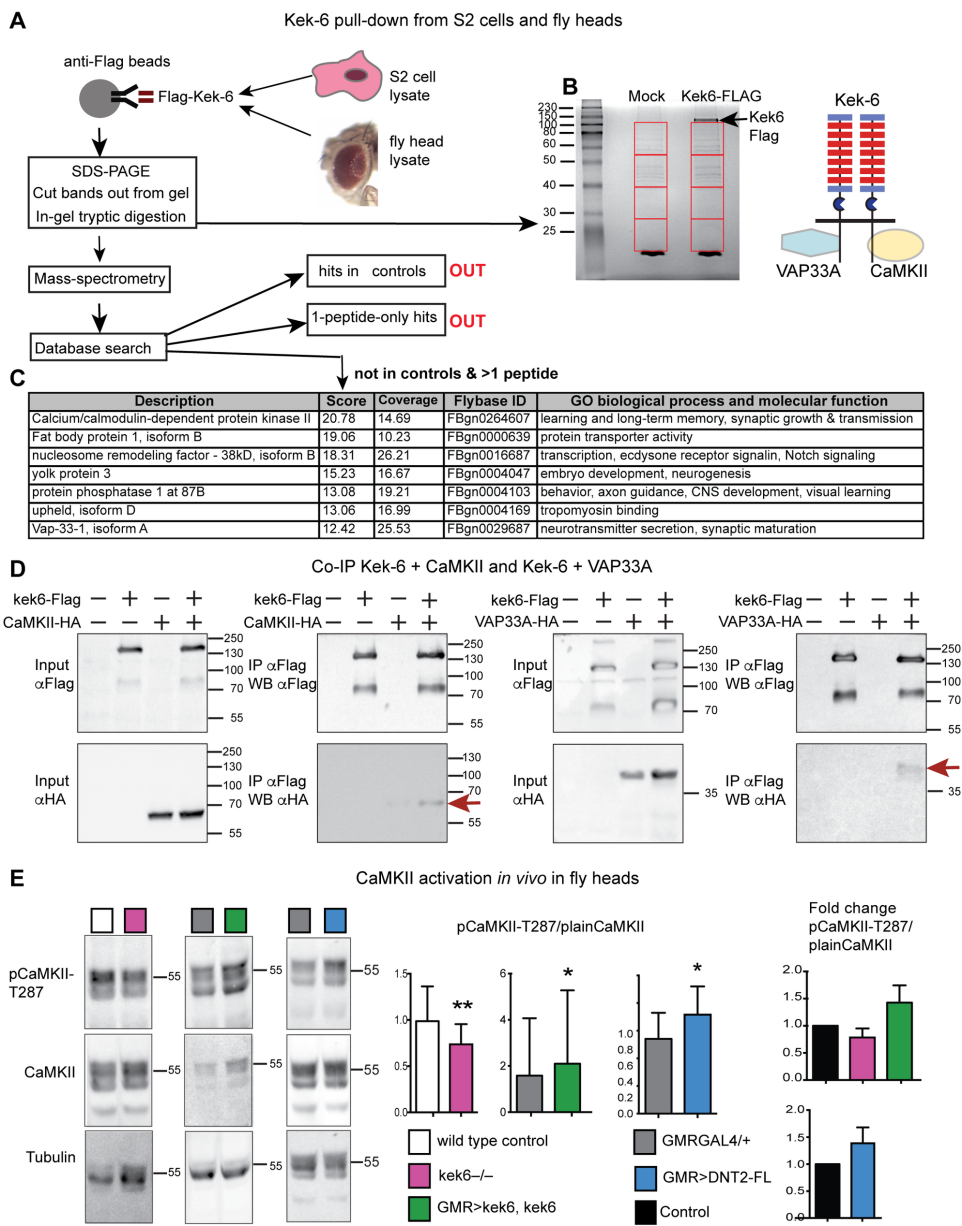
Kek-6 physically interacts with synaptic factors. (A) Diagram of pull-down workflow and (B) coomassie-stained gel showing proteins from S2 cell or adult fly head lysate bound to Kek-6-Flag or mock control. Red boxes indicate fractions used for mass spectrometry. Hits that appeared in mock controls, or with only one peptide, were dis-regarded. (C) Selection of candidate interacting proteins, see S2 and S3 Tables. (D) Co-immunoprecipitations from co-transfected S2 cells: precipitating Kek-6-Flag brought down CaMKII-HA and VAP33A-HA. IP: immunoprecipitation, WB: western blot. (E) Western blots with anti-activated pCaMKII^T287^, non-phosphorylated plain CaMKII and Tubulin, from adult fly heads, GMRGAL4 drives expression in retina. Graphs show pCaMKII^T287^ levels normalized over non-phosphorylated CaMKII. Student paired t-tests. *p<0.05, **p<0.01, n = 5–9 replicates, see S1 Table.

To find out how Kek-6 might function, we carried out pull-down assays to isolate candidate factors binding its intracellular domain. S2 cells were transfected with *kek6-Flag*, and anti-Flag coated beads were used to expose Kek-6 to cell lysates from either S2 cells or wild-type adult fly heads, and bound proteins were isolated by SDS-PAGE followed by mass spectrometry (Fig 8A and 8B). Candidates were identified as proteins present in Kek6-Flag samples and absent from non-transfected mock controls, and if identified from multiple peptides (S2 and S3 Tables). Prevalent amongst these were proteins involved in vesicle trafficking, axonogenesis, dendrite morphogenesis and synaptic function (Fig 8C). Amongst the top hits were CaMKinase II identified from fly heads, and VAP33A from both S2 cells and heads (Fig 8C). CaMKII functions both as a kinase and a scaffolding protein, to promote structural synaptic plasticity. Post-synaptically, it phosphorylates and recruits AMPAR and NMDAR to the post-synaptic density, leading to post-synaptic potentiation, and pre-synaptically it localizes to active zones and phosphorylates Synapsin and other SNARE complex proteins, triggering neurotransmitter release [48–51]. VAP33A is a Vamp Associated Protein, with evolutionarily conserved functions in exocytosis and vesicle trafficking, at synapses and in wider contexts [52]. To validate these two candidates as downstream effectors of Kek-6, we carried out co-immunoprecipitations. We co-transfected S2 cells with Kek6-Flag and either CaMKII or VAP33A tagged with HA. Precipitating Kek-6-Flag, brought down CaMKII-HA (Fig 8D). Similarly, precipitating Kek-6-Flag also brought down VAP33A-HA (Fig 8D). Thus, Kek-6 can bind CaMKII and VAP33A.

To validate the functional relationship between Kek-6 and CaMKII *in vivo*, we asked whether altering Kek-6 function would affect CaMKII activation in brain. We tested for the constitutively active state of CaMKII, corresponding to phosphorylation at Thr286—T287 in *Drosophila*—using antibodies that detect pCaMKII^T287^. In the heads of *kek6*^*34*^*/Df(3R)ED6361* mutant adult flies the relative levels of pCaMKII^T287^, normalised over non-phosphorylated CaMKII, decreased (Fig 8E, S1 Table). Conversely, over-expression of *kek-6* in retina (with *GMRGAL4*) increased CaMKII phosphorylation (Fig 8E, S1 Table). Over-expressing *DNT2-FL* had the same effect (Fig 8E). Thus, Kek-6 is required for CaMKII activation, and both Kek-6 and DNT2 can activate CaMKII downstream.

**Fig 9:**
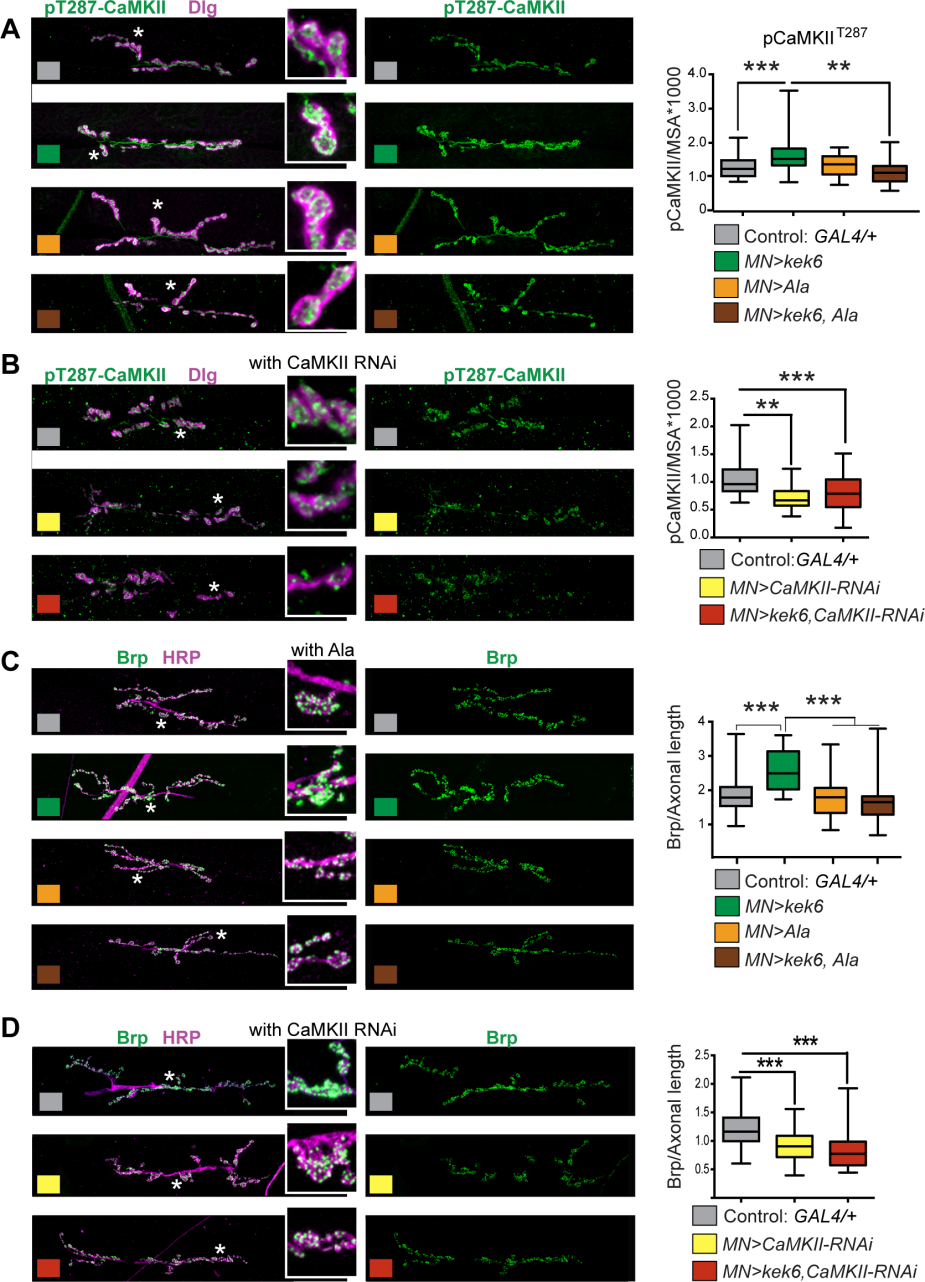
Kek6 activates CaMKII at the NMJ. NMJs from A3-4 muscle 6/7 (left), and box-plot graphs (right), NMJs, labeled with anti-pCaMKII^T287^ for the constitutively active form; anti-Dlg for post-synaptic boutons; anti-HRP for pre-synaptic axonal terminal length; anti-Brp for active zones. Brp and pCaMKII^T287^ were quantified automatically with DeadEasy Synapse. Higher magnification details are of areas indicated by asterisks. (A, B) Over-expression of *kek-6* in motoneurons increased pCaMKII^T287^ levels, which was rescued with the over-expression of the CaMKII inhibitor Ala (A) *or* CaMKII RNAi knock-down (B) pre-synaptically. Kruskal-Wallis p<0.0001 and **p<0.01, ***p<0.001post-hoc Dunn for both graphs. (C,D) CaMKII inhibition with Ala (C) or knock-down with CaMKII-RNAi (D) rescued the increase in active zones caused by *kek-6* over-expression. (C) One-way ANOVA p<0.000 and ***p<0.001 post-hoc Bonferroni, (D) Kurskal-Wallis p<0.0001 and **p<0.01, ***p<0.001 post-hoc Dunn’s. See S1 Table. N = 25–43 hemisegments. MN = motoneurons. Genotypes: (A-D) Control: *w; D42GAL4/+*. (A, C) *w; D42GAL4/UASkek6RFP*. *w; D42GAL4/UASAla; w; D42GAL4/UASkek6RFP UASAla*. (B, D) *D42GAL4/UASCaMKIIRNAi*. *w; D42GAL4/UASkek6RFP UASCaMKIIRNA*i.

Next we asked whether CaMKII and VAP33A might function downstream of Kek6 at the NMJ. Unfortunately, we were not able to get antibodies to inactive CaMKII to work reliably at the NMJ for normalisation, so we visualised constitutively activated CaMKII with anti-pCaMKII^T287^ and quantified it automatically, using DeadEasy Synapse [39]. DeadEasy Synapse detected the increase in pCaMKII caused by the over-expression of activated CaMKII^T287^ in motoneurons (*D42>CaMKII*^*T287D*^, S5A and S5C Fig). Over-expression of *CaMKII*^*T287*^ pre-synaptically in motoneurons increased axonal length, Ib bouton number and active zone density (S5A and S5C Fig, S1 Table). Conversely, inhibiting CaMKII only pre-synaptically by over-expressing the CaMKII phosphorylation inhibitor *Ala* [53] in motoneurons resulted in smaller NMJs and reduced active zones (S5A and S5B Fig, S1 Table), as previously reported [54]. Thus we asked whether Kek-6 influenced CaMKII at the NMJ. Pre-synaptic over-expression of *kek-6* in motoneurons increased pCaMKII^T287^ levels (*D42>kek6* Fig 9A, S1 Table). This increase was rescued by the pre-synaptic over-expression of *Ala* together with *kek-6 (D42>kek6*, *Ala)* showing that this phenotype was specific (Fig 9A, S1 Table). However, pre-synaptic over-expression in motoneurons of *Ala* alone *(D42>Ala)* did not result in a detectable reduction in pCaMKII in this case (Fig 9A, S1 Table). To try an alternative approach, we knocked-down pre-synaptic *CaMKII* expression with RNAi in motoneurons, and this decreased overall pCaMKII^T287^ levels, causing a stronger effect than Ala (*D42>CaMKII-RNAi*, Fig 9B S1 Table). Like Ala, pre-synaptic *CaMKII-RNAi* knock-down together with *kek-6* over-expression in motoneurons also rescued pCaMKII^T287^ levels, and reduced them further than controls (*D42>kek6*, *CaMKII-RNAi*, Fig 9B, S1 Table). This demonstrates that CaMKII functions downstream of Kek-6 in motoneurons. Together, these data show that Kek-6 function results in the activation of CaMKII pre-synaptically.

**Fig 10:**
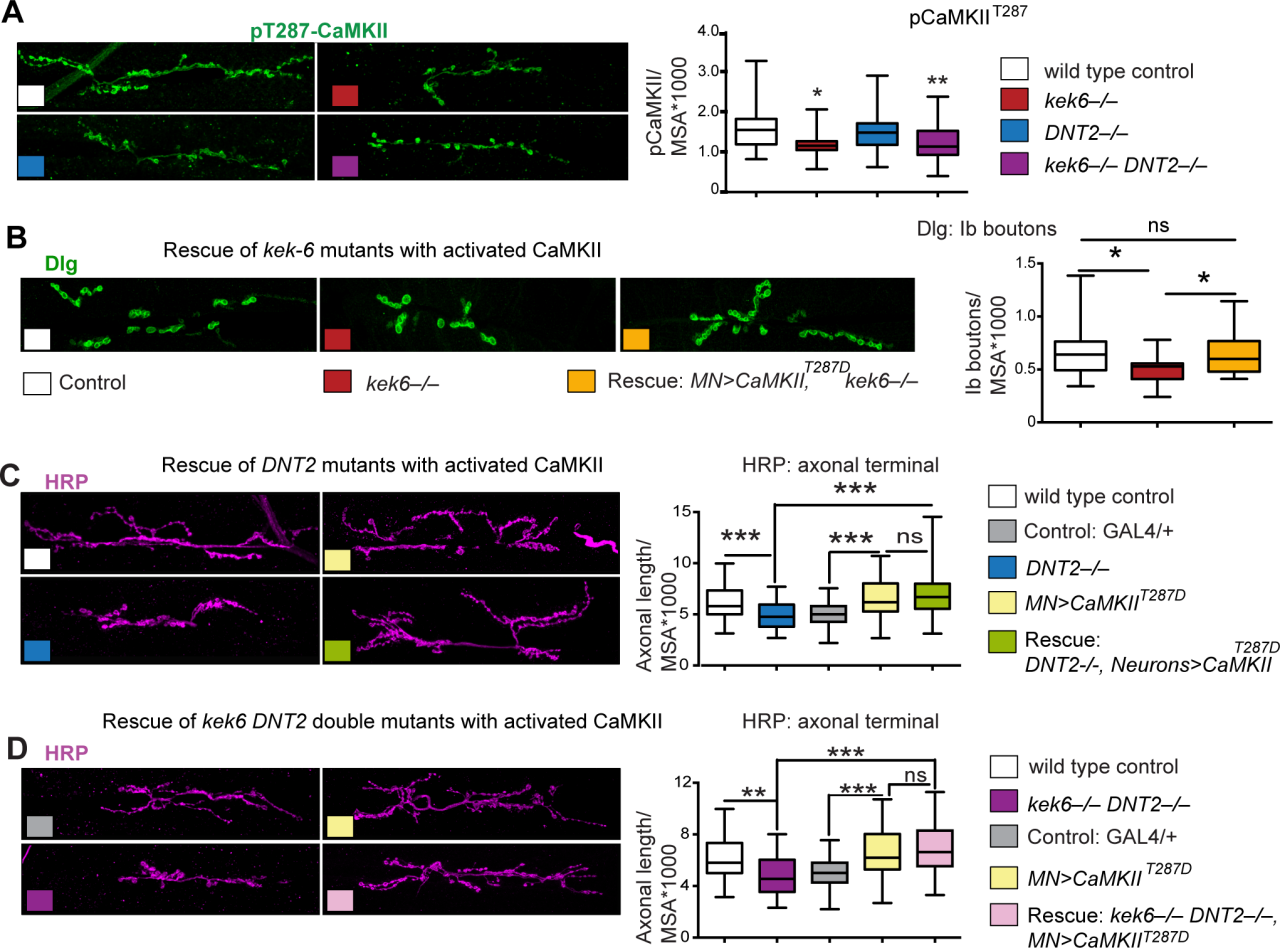
CaMKII functions downstream of Kek6 and DNT2 at the NMJ. Confocal images showing A3-4 muscle 6/7 NMJs, labeled as in Fig 9. (A) Reduced pCaMKII^T287^ levels in *kek-6* mutants, and *kek-6 DNT2* double mutants. Kurskal-Wallis p = 0.0009, and post-hoc Dunn. (B-D) Over-expresison of activated *CaMKII*^*T287D*^ in motoneurons rescued the NMJ phenotypes of (B) *kek-6* mutants, Kruskal-Wallis p = 0.0081, post-hoc Dunn; (C) *DNT2* mutants, Kruskal-Wallis p = 0.0001, post-hoc Dunn, and (D) *kek-6 DNT2* double mutants. Welch ANOVA p<0.000, post-hoc Games-Howell. *p<0.05, **p<0.01, ***p<0.001 see S1 Table. N = 23–75 hemisegments. Genotypes: MN = motoneurons, *D42GAL4;* Neurons: *elavGAL4*. Different neuronal drivers were used due to genetic constraints. Control: wild-type: *yw/+;* grey boxes: *D42GAL4/+*. (A) Mutant genotypes as in Fig 4; Rescues: (B) *w;UASCaMKII*^*T287D*^*/+; D42GAL4 kek6*^*34*^*/ Df(3R)6361*. (C) *w;UASCaMKII*^*T287D*^*/+; elavGAL4 Df(3L)6092/DNT2*^*37*^. (D) *w; UASCaMKII*^*T287D*^*/Toll-7GAL4; kek6*^*34*^*Df(3L)6092/ Df(3R)6361 DNT2*^*37*^.

Pre-synaptic activated CaMKII localizes active zones [49,55], so next we asked whether CaMKII was required for the increased active zones caused by *kek-6* gain of function. Pre-synaptic CaMKII inhibition with Ala or knock-down with RNAi in motoneurons decreased active zones (S5A and S5B Fig and Fig 9D, S1 Table) and over-expression of *activated CaMKII* in motoneurons increased active zones (S5A and S5C Fig, S1 Table). Remarkably, pre-synaptic over-expression in motoneurons of *Ala* or *CaMKII-RNAi* together with *kek-6*, rescued the increase in active zones caused by *kek-6* over-expression (Fig 9C and 9D, S1 Table). These showed that CaMKII is required downstream of Kek-6 for active zone formation. Furthermore, in *kek6*^*–/–*^mutants and *kek6*^*–/–*^*DNT2*^*–/–*^double mutants the levels of pCaMKII^T287^ decreased (Fig 10A, S1 Table), showing that Kek-6 is required for CaMKII activation at the NMJ. We found no significant effect of *DNT2* loss or gain of function on pCaMKII^T287^ levels at the NMJ. Importantly, over-expressing activated *CaMKII*^*T287D*^ pre-synaptically rescued the phenotypes of decreased axonal length and reduced Ib boutons of *kek6*^*–/–*^(Fig 10B) and *DNT2*^*–/–*^(Fig 10C) single mutants, and *kek6*^*–/–*^*DNT2*^*–/–*^double mutants (Fig 10D, S1 Table). This means that the mutant phenotypes were caused, at least partly, by decreased CaMKII activation. Together, these data demonstrate that Kek-6 and DNT2 function in concert upstream of CaMKII to regulate NMJ size and active zones.

**Fig 11:**
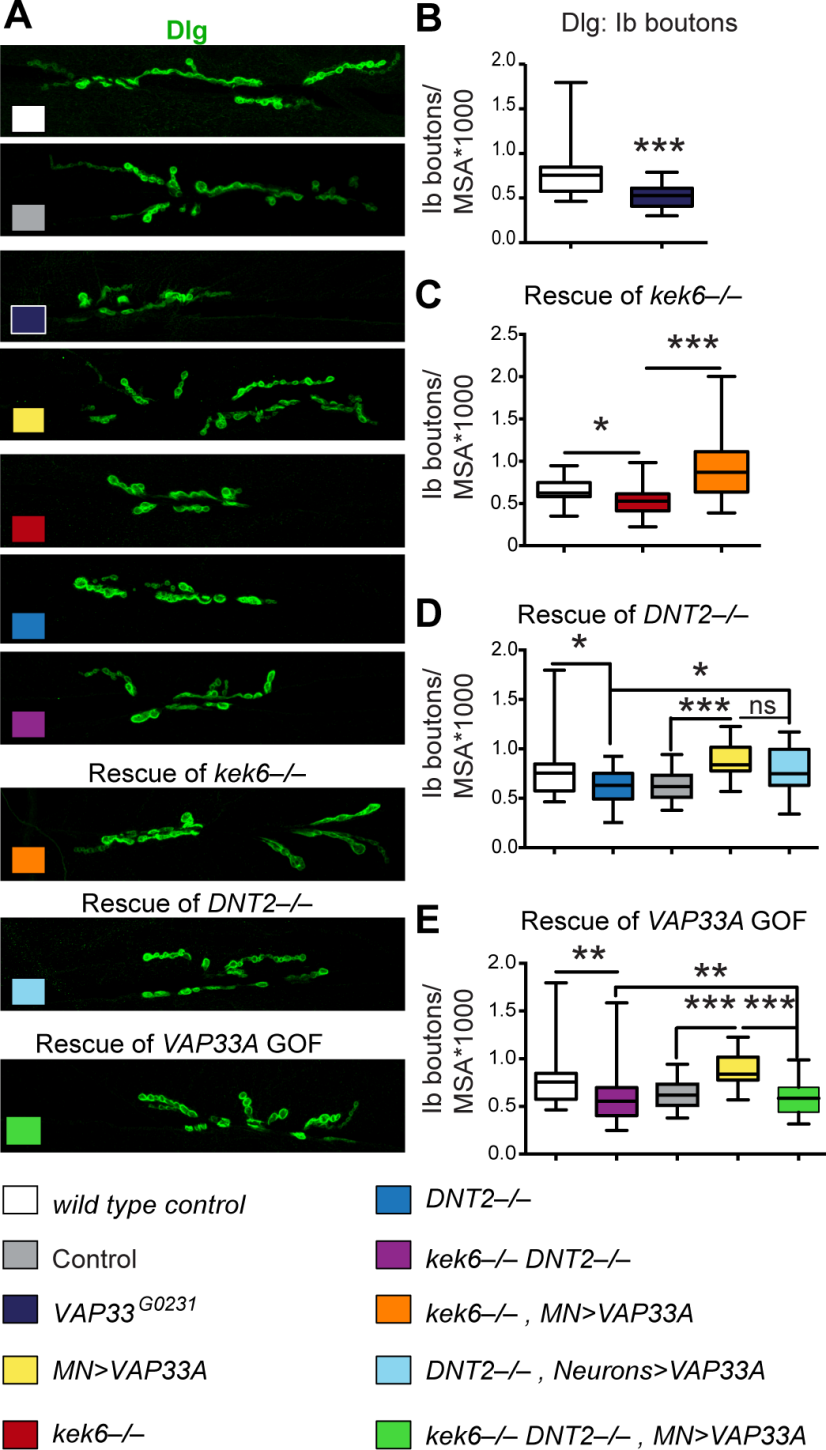
VAP33A functions downstream of Kek-6. (A) Confocal images of NMJs from segments A3-4, muscle 6/7. (B-E) Box-plot graphs. (B) *VAP33A*^*G0231*^ mutants have reduced bouton number, Mann-Whitney U test ***p<0.001. (C,D) Pre-synaptic over-expression of *VAP33A* rescues bouton number in (C) *kek-6*^*–/–*^mutants and (D) *DNT2*^*–/–*^single mutants, Kruskal-Wallis p<0.0001 and *p<0.05, ***p<0.001 post-hoc Dunn for both. (E) *kek-6*^*–/–*^*DNT2*^*–/–*^double mutants rescue the bouton number phenotype caused by *VAP33A* gain of function, Kruskal-Wallis p<0.0001 and **p<0.01, ***p<0.001 post-hoc Dunn. See S1 Table. N = 23–48 hemisegments. MN = motoneuron, *D42GAL4* (D) or *Toll-7GAL4* (E); Neurons = *elavGAL4*. Rescue genotypes: (C) *UASVAP33A/+; D42GAL4 kek6*^*34*^*/Df(3R)6361*. (D) *UASVAP33A/+; elavGAL4 Df(3L)6092/DNT2*^*37*.^. (E) *UASVAP33A/Toll-7GAL4; kek6*^*34*^*Df(3L)6092/ Df(3R)6361 DNT2*^*37*^.

To test the functional link between Kek-6, DNT2 and VAP33A, we used genetic epistasis. Loss of function *VAP33A*^*G0231*^ mutants had fewer Ib boutons (Fig 11A and 11B), and pre-synaptic over-expression of *VAP33A* increased Ib bouton number (Fig 11A, 11D and 11E, S1 Table), as previously reported[52]. These phenotypes were also shared with alterations in *kek-6* and *DNT2* levels, consistent with common functions. Importantly, neuronal over-expression of *VAP33A* rescued bouton number in *kek-6*^*–/–*^(Fig 11A and 11C) and *DNT2*^*–/–*^(Fig 11A and 11D) single mutants (S1 Table). Thus, VAP33A functions downstream of Kek-6 and DNT2. Interestingly, *kek-6*^*–/–*^*DNT2*^*–/–*^double mutants rescued the increase in Ib boutons caused by *VAP33A* over-expression, restoring bouton number down to control levels (Fig 11A and 11E, S1 Table). This suggests that VAP33A may be required both for pre-synaptic vesicle release downstream of Kek-6, and post-synaptic secretion of DNT2.

To conclude, altogether these data show that CaMKII and VAP33A function downstream of DNT2 and Kek-6 in motoneurons.

## Discussion

**Fig 12:**
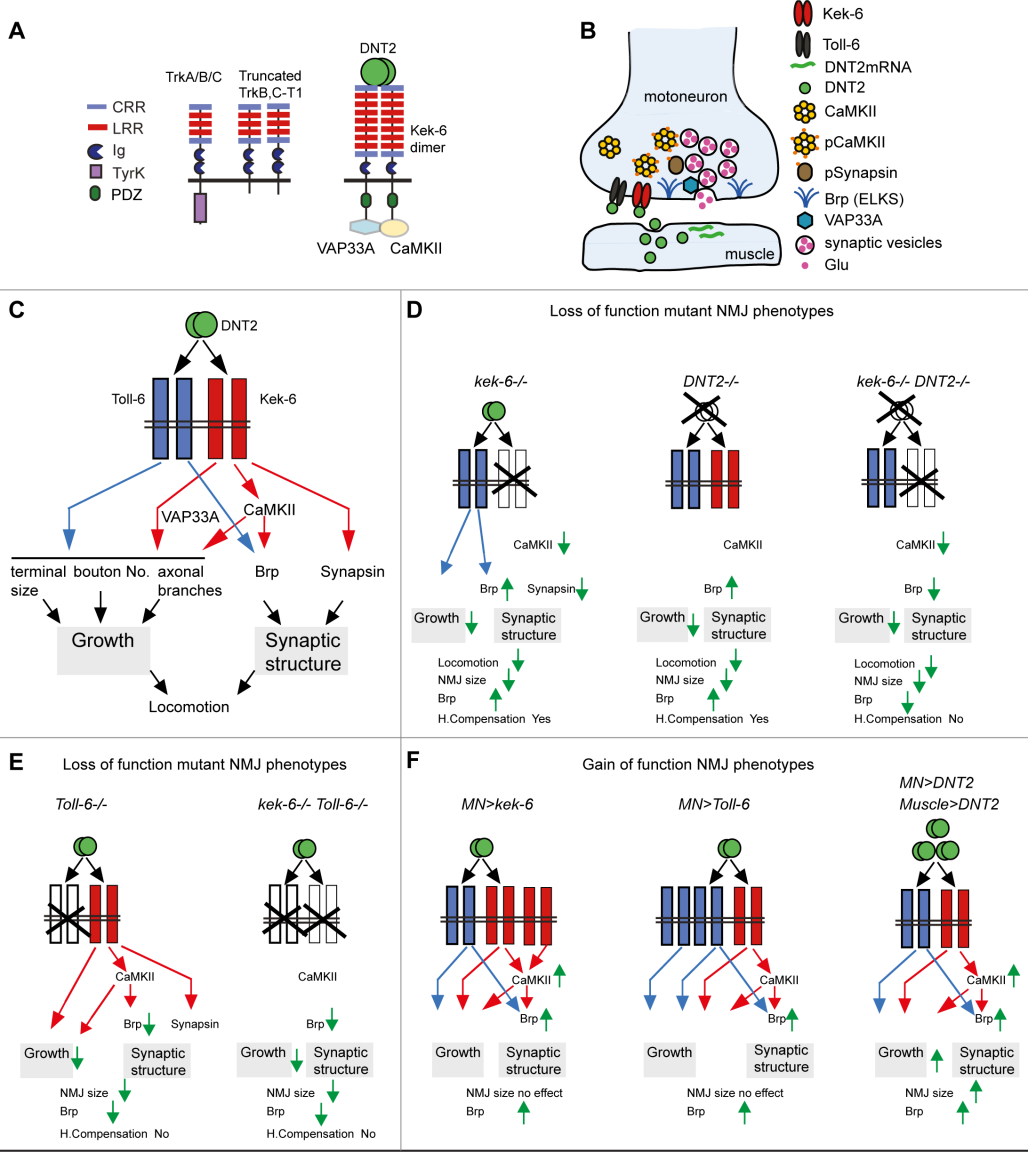
Retrograde DNT2 binds pre-synaptic Kek-6 activating CaMKII and regulating structural synaptic plasticity. (A) Illustration of Kek-6 compared to Trk isoforms. DNT2 binds Kek-6, which functions via CaMKII and VAP33A downstream. (B) Pre-synaptic motoneuron terminal at the NMJ: DNT2 is produced at the muscle and secreted, binds pre-synaptic Kek-6, functioning via CaMKII and VAP33A downstream. DNT2 also binds Toll-6 which can interact with Toll-6. (C) The concerted functions of DNT2 and its two receptors Kek-6 and Toll-6 regulates NMJ growth and synaptic structure. Kek-6 functions via CaMKII and VAP33A downstream, the mechanism downstream of Toll-6 at the NMJ has not been investigated in this work. Red arrows: positive regulation by Kek-6; blue arrows: positive regulation by Toll-6. (D-F) Summary of the experimental evidence provided, green arrows indicate up- or down-regulation as a result or loss or gain function genotypes. Altering the levels of DNT2, Kek-6 and Toll-6 affects locomotion, NMJ growth and synaptic structure. Importantly, loss of both kek-6 and Toll-6 prevents homeostatic compensation of active zones, and whereas gain of function for kek-6 or Toll-6 is not sufficient to increase NMJ size, over-expression of DNT2 can. The data suggest that Kek-6 and Toll-6 function in concert as a receptor complex for DNT2, to regulate structural synaptic plasticity.

Drosophila homologues of the Trk receptor family had long been sought. This was important to find fundamental principles linking structure and function in any brain. Here, we show that Keks are Trk-family receptors lacking a TyrK, for *Drosophila* neurotrophin ligands (DNTs) (Fig 12A). Kek-2, -5 and -6 are expressed in the CNS, and genetic rescues, a signaling assay and co-immunoprecipitations demonstrate that they bind DNTs promiscuously. For in vivo analyses we focused on Kek-6. We demonstrate that motoneurons express *kek-6* pre-synaptically, and Kek-6 binds DNT2, which is produced by the muscle from post-synaptic boutons (Fig 12B). *kek-6*^*–/–*^and *DNT2*^*–/–*^share mutant phenotypes, and using genetics we demonstrate they are functionally linked in vivo. Kek-6 can interact physically with the alternative DNT2 receptor, Toll-6. Most likely Kek-6 and Toll-6 function as a DNT2 receptor complex (Fig 12C). We show that: (1) The concerted functions of Toll-6 and Kek-6 are required to regulate NMJ growth. All single mutants–*kek-6*^*–/–*^, *Toll-6*^*–/–*^*and DNT2*^*–/–*^–decreased NMJ size and complexity (Fig 12D and 12E), but only over-expression of DNT2 could induce a dramatic increase in NMJ size (Fig 12F). This shows that the compound activation of both receptors by DNT2 has a stronger effect on NMJ growth that activating each alone (Fig 12F). (2) Kek-6 and Toll-6 can each regulate active zone formation via alternative pathways, as Kek-6 can promote active zone formation independently of Toll-6 (Fig 12C). The ability of both Kek-6 and Toll-6 to regulate active zones enables homeostatic compensation in single mutants (Fig 12D). We show that Kek-6 functions by recruiting CaMKII and VAP33A to a pre-snaptic downstream complex and induces CaMKII activation. Epistasis analysis demonstrated that Kek-6 functions in motoneurons downstream of DNT2 and upstream of CaMKII and VAP33A to promote both NMJ growth, and active zone formation (Fig 12C). We conclude that at the NMJ, Kek-6 is a pre-synaptic receptor for DNT2, regulating structural synaptic plasticity (Fig 12A–12C).

### DNT2 is a retrograde factor at the NMJ

Despite abundant evidence that retrograde signals and positive feedback loops regulate structural synaptic plasticity at the *Drosophila* glutamatergic NMJ, the identification of retrograde factors had been scarce [45,56]. This created a void for the general understanding of structural synaptic plasticity across different brain types. BDNF is a key retrograde ligand in mammals, and together with its receptor TrkB they form a positive feedback loop that reinforces synaptic function [1,4]. We have shown that the neurotrophin DNT2 is a retrograde ligand for Kek-6 (Fig 12B). *DNT2* is expressed in muscle and both its receptors *kek-6* and *Toll-6* are expressed in motoneurons. Over-expression of *DNT2* in muscle leads to its distribution in pre-synaptic boutons; over-expression of *DNT2* in muscle induces NMJ growth and active zone formation pre-synaptically; *DNT2* mutants have smaller NMJs; pre-synaptic over-expression of either *kek-6*, *CaMKII* or *VAP33A* can rescue the *DNT2* loss of function phenotype; and conversely, *kek6* loss of function rescues the phenotype caused by *DNT2* over-expression in muscle. Thus, DNT2 is an evolutionarily conserved retrograde factor that regulates structural synaptic plasticity in *Drosophila*. This finding will have an impact bridging the universal understanding of synaptic plasticity and potentiation.

### Keks are the Trk-family receptors in Drosophila

We demonstrate that *keks* are the *Drosophila Trk*-family homologues (Fig 12A). Previous searches for Trk-family receptors in fruitflies had focused on the Tryrosine Kinase domain, and did not identify any bona fide candidates [14–16,18]. Subsequent proteomic analyses confirmed the absence of full-length Trk receptors with a conserved tyrosine kinase in fruitflies [19–21]. Since those earlier searches, Trks in mammals have been found to encode multiple isoforms, most of which lack the tyrosine kinase domain [6,57]. Human TrkB has 100 isoforms, which produce 36 different proteins, of which four are abundant. Importantly, the most abundant isoform in the adult human brain is truncated TrkB-T1, which lacks the tyrosine kinase [6,57]. Paradoxically, the function of TrkB-T1 in neurons in the mammalian brain is unknown. Our findings suggest that truncated Trk family receptors could have an evolutionarily conserved function regulating structural synaptic plasticity.

We demonstrate that Kek-6 can activate CaMKII downstream (Fig 12B and 12C). However, we do not yet know how this may come about. CaMKII activation depends on the intracellular increase in Ca^2+^ levels, either through Ca^2+^ channels, or Ca^2+^ stores through the PLC and IP3 pathways [50,55]. In mammals, TrkB-T1 regulates Ca^2+^ levels in glia and in the heart, two contexts expressing high levels of TrkB-T1 and no TrkB-FL [58–60]. However, the mechanism by which TrkB-T1 raises Ca^2+^ is unknown [58,59]. Furthermore, whether this results in the activation of CaMKII is unclear or may be context dependent [58,60]. CaMKII activation in neurons could depend on the increase in Ca^2+^ with neuronal activity. A potential link of Kek-6 to neuronal activity was revealed by the increase in ghost boutons upon alterations in *kek-6* levels (S4 Fig). Furthermore, *kek-2* expression is modulated by neuronal activity, appropriate Kek-2 levels at the NMJ are required for normal synaptic structure, and DNT2 can modulate the Na+/K+ ATPase [27,61]. Thus, Kek-6 could function via a PLCβ pathway or by influencing membrane channels, to increase Ca^2+^ levels and induce CaMKII activation.

The finding that Trk-like receptors can regulate neuronal plasticity independently of kinase signaling is very important. Activation of PLCγ by TrkB TyrK signaling is generally seen as the key mechanism of Trk-depedent plasticity [1,5]. Yet CaMKII is necessary and sufficient for synaptic structural plasticity, LTP and long-term memory, and has wide functions in synaptic organization and homeostasis [49–51]. CaMKII can function as a frequency detector, and cause long lasting changes in synaptic strength, structure and brain plasticity [50]. Thus, the regulation of CaMKII and Ca^2+^ by truncated Trk-family receptors and Keks means that they could regulate structural synaptic plasticity independently of the canonical TyrK-dependent PLCγ pathway.

Keks are not identical to truncated Trk receptors and may carry out further functions that could be implemented via other routes in mammals. TrkB-T1 has a very short intracellular domain, whereas Kek-2, -5 and -6 have longer intracellular fragments that include a PSD95/Dlg/ZO1 (PDZ) motif [22,24,25]. PDZ domains are involved in scaffolding, and assembly of post-synaptic complexes that regulate the size and strength of synapses [62]. Through its PDZ domain, Kek-6 might recruit synaptic partners and/or Ca^2+^ channels. Interestingly, truncated TrkC-T1 binds the PDZ-containing protein Tamalin to induce cellular protrusions via Rac[63]. Exploring further signaling mechanisms of Keks vs. truncated Trks could uncover novel mechanisms of synaptic plasticity, perhaps also in the human brain.

### Novel mechanism of structural synaptic plasticity involving concerted functions of DNTs, Trk and Toll receptor families

LIG proteins and truncated Trk isoforms can function as ‘dominant negative’ co-receptors or ‘ligand sinks’. TrkB-T1 can form dimers with TrkB-FL and abrogate signaling, and bind BDNF rendering it unavailable to TrkB-FL [6,7]. This is thought to modulate kinase-signaling levels by TrkB [64]. Similarly, in *Drosophila*, Kek-5 is an inhibitor of BMP signaling [26] and Kek-1 an inhibitor of EGFR signaling [65]. Thus, each Kek could function as a specific inhibitor of different signaling pathways. By contrast, our evidence shows that Kek-6 does not function as an inhibitor of Toll-6. Instead, it indicates that Kek-6 and Toll-6 function in concert to regulate NMJ structure and growth (Fig 12C). Firstly, *kek-6*^*–/–*^, *Toll-6*^*–/–*^and *DNT2*^*–/–*^single mutants, and *kek-6*^*–/–*^*Toll-6*^*–/–*^double mutants, all have smaller NMJs, ruling out antagonistic functions between the two receptor types (Fig 12D and 12E). Secondly, whereas over-expression of *kek-6* or *Toll-6* alone did not increase NMJ size, over-expression of *DNT2* did, showing that the activation of both receptor types at once has a stronger effect than each alone (Fig 12F). This also rules out an inhibitory function. Thirdly, Toll-6 is required for active zone formation, and over-expression of either *Toll-6* or *kek-6* increases active zones (Fig 12C–12F). This implies that Kek-6 does not inhibit Toll-6. Instead, DNT2 can modulate the homeostatic compensation of active zones, via its two receptors (Fig 12D and 12E). Indeed, compensation fails in *kek6*^*–/–*^*DNT2*^*–/–*^and *kek-6*^*–/–*^*Toll-6*^*–/–*^double mutants (Fig 12D and 12E). The data suggest that Kek-6 and Toll-6 influence synaptic structure via alternative, independent pathways. Toll-6 generally functions in neurons via NFκB and JNK [36], and at the NMJ Toll-6 and Toll-8 regulate structural synaptic plasticity through Sarm, JNK and dFOXO [37,43]. Here, we show that instead, Kek-6 functions via CaMKII and VAP33A to regulate active zones and synaptic structure (Fig 12B and 12C). Kek-6 functions independently of Toll-6, as it can induce active zone formation in *Toll-6* mutants; the induction of active zones downstream of Kek-6 depends on CaMKII; and there is no evidence that Toll-6 can activate CaMKII on its own. Furthermore, Kek-6 also affects other synaptic factors, like Synapsin. We have shown that Kek-6 and Toll-6 can interact physically, thus could form a receptor complex for DNT2. We conclude that DNT2 regulates NMJ size and active zone formation, by engaging a receptor complex that drives distinct parallel pathways downstream (Fig 12C).

An intriguing observation is that DNTs can bind various Tolls [38] and Keks (this work) promiscuously, and similarly multiple Tolls can bind Kek-6. The biological significance of such promiscuity is not understood. Whether this reflects redundancy will depend on the spatial, cell-type specific and temporal distribution of each ligand and receptor. Redundancy can serve to compensate for deficits, thus conferring robustness to the CNS and resulting behaviour. For instance, homeostatic compensation of active zones vs. NMJ size, which was also observed in Toll-8 mutants[37]. Either way, our findings show that DNTs, Keks and Tolls working together constitute a novel molecular mechanism for synaptic structural plasticity and homeostasis. Whether this could be mirrored by the mammalian NTs, Truncated Trks and TLRs, will be intriguing and important to find out.

To conclude, evolution may have resolved how to implement structural synaptic plasticity through distinct mechanisms in fruit-flies and humans, or perhaps common molecular principles are shared by both, even if not in every detail. We found that in fruitflies, truncated-Trk-like receptors encoded by the Keks bind neurotrophin ligands to regulate structural synaptic plasticity via CaMKII and VAP33A, and the receptor complex also includes Tolls. It is compelling to consider whether such a non-canonical mechanism of neuronal plasticity, downstream of neurotrophins and Trk receptors but independently of kinase signaling, may also operate in humans, as it could uncover novel mechanisms of brain function and brain disease.

## Materials and methods

### Genetics

**Mutants:** *Df(3R)ED6361* lacks the *kek-6* locus (Kyoto Stock Centre), *Df(3L)6092*, *Df(3L)ED4342*, *DNT1*^*41*^ and *DNT2*^*e03444*^ are described in[35], *Df(3L)BSC578* lacks the *Toll-6* locus (Bloomington). Mutant null alleles *kek6*^*34*^ and *kek6*^*35*^, and *DNT2*^*37*^, were generated by FRT mediated recombination of the PiggyBac insertion lines [46]: for *Kek6*: *PBac[RB]kek6*^*e000907*^ and *PBac[WH]kek6*^*f05733*^; for *DNT2*: *PBac[RB]spz5*^*e03444*^ and *PBac[WH]Shab*^*f05893*.^. Mutants were selected using genetics and PCR, using primers as recommended in [46]. *kek6*^*34*^ is a 41.9 kb deletion that just removes the coding region for *kek6;* DNT2^37^ is a 27 kb deletion that removes the ATG and first exon, most likely resulting in no protein production [36]. *VAP33A*^*G0231*^ is semi-lethal *P-lac* insertion allele from Bloomington (*w P{lacW]VAP33A*^*G0231*^). *Toll-6* mutants used have been described in [38]. **GAL4 lines:** *kek5GAL4* line *P[GawB]NP5933* (from BSC); *w;;elavGAL4* drives expression in all neurons (insertion on the third chromosome); *Toll-6-GAL4* was generated by RMCE from *MIMICToll-6*^*MIO2127*^ and *kek-6-GAL4* from *MIMICkek-6*^*MI13953*^*; w;;D42GAL4* and *w;Toll-7GAL4* drive expression in motoneurons and have been previously described[38]; *w; MhcRFP MhcGAL4* drives expression in muscle (Bloomington); *w; GMRGAL4* drives expression in retina. **UAS lines**: to drive expression of each of the *keks* UAS-lines were made as described below. *w;;UAS-DNT2-FL* expresses full length untagged DNT2; *w;;UASDNT2-CK* expresses the mature form of DNT2, i.e. signal peptide+cystine-knot domains; *w; UAS-DNT2-FL-GFP* expresses full-length DNT2 tagged at the COOH end, and were made as described below. *w; 10xUASmyr-Td-Tomato* is membrane tethered (gift of B. Pfeiffer); *w;* UASFlyBow (gift of I.Salecker); *w;UASCaMKII*^*T287D*^ expresses constitutively active CaMKII and *w;UASAla* expresses the CaMKII inhibitor (gifts of J. Hodge)*; UASCaMKII-RNAi*: *y sc v; P{y[+ v+*, *TRiP*.*GL00237}attP2/TM3*, *Sb* (BSC); y[1] w[*]; P{w[+mC] = UAS-FLAG.Vap-33-1.HA}2 (Bloomington). Double mutant lines were generated by conventional genetics. **MIMIC-GFP lines:** *y w; MIMICkek6*^*MI13953*^ and has a GFP insertion in the coding region (BSC). All stocks were balanced over *SM6aTM6B* or *TM6B* to identify balancer chromosomes, and all were generated from a *yw* or *w* mutant background. In all figures, controls are: (1) F1 from *yw x Oregon*; (2) *y w; GAL4/+*

### Molecular cloning

Full length cDNAs for *kek1*, *2*, *3*, *4*, *5* and *6* were obtained either from cDNA clones (*kek1*, *SD01674*; *kek2*, *NB7*), by PCR from cDNA libraries (gift of G. Tear; LD: *kek5;* GH: *kek3*; *kek6*), or by reverse transcription PCR from larvae (*kek4*), using primers designed for Gateway cloning into the pDONR plasmid: *kek1* forward: GGGGACAAGTTTGTAC AAA AAA GCA GGC TCA TCC AGG AAA **ATG** CAT ATC A and reverse: GGGGACCAC TTT GTA CAA GAA AGC TGG GTA GTC AGT TCT TGG TTT GGT TT; *kek2*: forward: GGGGACAAGTTTGTAC AAA AAA GCA GGC TCA **ATG** AGT GGT CTG CCA ATC T and reverse: GGGGACCAC TTT GTA CAA GAA AGC TGG GTA AAT GTC GCT GGT TTC CTG GC; *kek3*: forward: GGGGACAAGTTTGTAC AAA AAA GCA GGC TCA TAT GCG **ATG** GCA GCG GGA A and reverse: GGGGACCAC TTT GTA CAA GAA AGC TGG GTA GCT CTT GAA AAT ATC CTG TC; *kek4*: forward: GGGGACAAGTTTGTAC AAA AAA GCA GGC TCA CTA GAC CTT CCG TTC CTT, and reverse: GGGGACCAC TTT GTA CAA GAA AGC TGG GTA TAT TGA GAT ATC AAC ACC AG; *kek5*: forward: GGGGACAAGTTTGTAC AAA AAA GCA GGC TAG CTA GAC GCA GAC TTA GAG and reverse: GGGGACCAC TTT GTA CAA GAA AGC TGG GTA GAC CTC GGT GCC ATC CTC GC; *kek6*: forward: GGGGACAAGTTTGTAC AAA AAA GCA GGC TCA **ATG** CAT CGC AGC ATG GAT C and reverse: GGGGACCAC TTT GTA CAA GAA AGC TGG GTA GAG CGA CAC GAA CTC GCC AG. *CaMKII* and *VAP33A* full-length cDNAs were cloned using the Gateway System first into *pDONR*, and then into *pAct5-attR-HA*.

For expression in flies under UAS/GAL4 control, cDNAs were subcloned to a Gateway *pUASt*-*attR*-*mRFP* destination vector for conventional transgenesis, injected by BestGene (www.thebestgene.com). For expression in S2 cells, they were subcloned into tagged *pAct* destination vectors *pAct5c*-*attR*-*mCFP*, *pAct5c*-*attR*-*FLAG* and *pAct5c*-*attR*-*HA*. Constructs used for expression of S2 cells of DNT1 and 2, Toll-6 and 7 were as previously described[38]. Chimaeric Kek-Toll-6 receptors were generated after analyzing the domain composition of the proteins using ProSite (ExPASy), PFAM, SMART, TMHMM and TMPred algorithms and PubMed data. Primers were designed to amplify the sequences that encode the extracellular and transmembrane domains of Kek3–6, including 15 amino acids C-terminal to the Kek transmembrane region, and the intracellular domain of Toll6, at halfway between the transmembrane region and the TIR domain. A unique enzyme site (BamHI or EcoRI) was included in the designed primers to join the *kek* and *Toll-6* sequences, and attB sites were introduced in the primers at the 5′ and 3′ ends of the chimaeric insert for Gateway cloning into *pAct5c-attR-3xHA* destination vector. Unfortunately, we were not able to generate *kek1-Toll-6* and *kek2-Toll-6* chimaeric receptor constructs. To clone chimaeric *kek3*,*4*,*5*,*6-Toll-6* receptor constructs, reverse primers at the juxtamembrane of *keks* were as follows: *kek3-Toll-6*: CGAT-GAATTC-AGGTACAGAGTTCCAGAGAC; *kek4-Toll-6*: CGCG-GAATTC-TTGCAAATAAGTGTGCTGGC; *kek5-Toll-6*: CTAT-GGATCC-GCTCATCATGGTGGTGTCCT; *kek6-Toll-6*: GTAT-GAATTC-ACGCCGGCCTTGTTGGCATG. *Toll-6* primers from chimaeras were: Forward primers from juxtamembraneCATG-GAATTC-AACTTCTGCTACAAGTCACC (compatible with *kek3*, *kek4* and *kek6* chimaeras) or CATG-GGATCC-AACTTCTGCTACAAGTCACC (compatible with *kek5* chimaera) and reverse from C terminus: GGGGACCAC TTT GTA CAA GAA AGC TGG GTC CGC CCA CAG GTT CTT CTG CT.

Un-tagged full-length DNT2 was cloned into the pUASt-attB vector by conventional cloning, DNT2-full length (DNT2-FL) was PCR-amplified from cDNA libraries, and cloned into pUASt-attB for ΦC31 transgenesis [66]. UAS-DNT2-FL-GFP was cloned by Gateway cloning into pUAS-GW-GFP, followed by conventional transgenesis.

Reverse Transcription PCR was carried out following standard procedures after mRNA straction from wandering larvae, using the following primers: (1) Control: *GAPDH*: GAPDH Fw TCACCACCATTGACAAGGC; GAPDH Rev:; CGGTAAGATCCACAACGGAG; (2) *DNT2*: DNT2 0.5kDa Rwd TGGAAACCCGCTCTTTGTCAG; DNT2 0.9kDa Rev:.TGATGAACTGCGATGTCGTCT. The genotypes of larvae tested were *y w* control, and transheterozygous mutants *DNT2*^*37*^*/Df(3L)6092*.

### Luciferase signaling in S2 cells

For Dif signaling assays using chimaeric kek-Toll-6 receptors, S2 cells stably transfected with *drosomycin-luciferas*e were maintained at 27°C in air in Insect-Xpress medium (Lonza) supplemented with penicillin/streptomycin/L-glutamine mix (Lonza) and 10% foetal bovine serum (Lonza). 1ml suspended cells were passaged every two days into 4ml fresh medium. 3x10^6^ cells in 2ml media were seeded per well of a 6-well plate 24 hours prior to transfection. Per well of experiment, 250μl serum-free media, 3μl TransIT-2020 (Mirus), 2μg of HA tagged chimaeric receptors *kek-toll6-HA* plus 1μg of *pAct-renilla-luciferase* were incubated at room temperature for 30 minutes, supplemented with 350μl serum-free media and added to aspirated cells. After 4 hours, transfection mixture was removed and 2ml supplemented medium added. All experiments were conducted 48 hours after transfection; for imaging membrane targeting of Kek-Toll6-HA protein S2 cells were starved for 6h prior to fixation.

For signaling assays, S2 cells were stimulated with 50nM/well of purified baculovirus DNT2 protein (generated as previously described[38]) and Dif signaling quantified by luminescence. DNT2 was added 48 hours after transfection and luminescence quantified 24 hours after DNT stimulation. Transfected and stimulated cells were pelleted from single wells, resuspended in 400μl media and separated into three 50μl aliquots in an opaque 96-well plate. 40μl of Firefly Luciferase Substrate (Dual-Glo Luciferase Assay System; Promega) was added per 50μl aliquot, incubated for 10 minutes, and luminescence measured using a Mithras LB 940 Multimode Microplate Reader (Berthold). 40μl Stop & Glo substrate (Dual-Glo Luciferase Assay System; Promega) was added to quench Dif signal and activate Renilla Luciferase. Renilla luminescence data was used to normalize Firefly Luciferase data.

### Co-immunoprecipitations

Coimmunoprecipitations were carried out as previously described[38], after transfecting standard S2 cells with the following constructs: (1) Co-IP Kek6-DNT2: *pAct5C-Pro-TEV6HisV5-DNT2-CK* and *pAct5C-Kek6-3xHA*; (2) Co-IP Kek6-DNT2 or Kek6-DNT1: *pAct5C-Kek6-3xHA*, *pAct5C-Pro-TEV6HisV5-DNT1-CK-CTD*, *pAct5C-Pro-TEV6HisV5-DNT2-CK;* (3) Co-IP Kek5-DNT2: *pAct5C-Kek5-3xFlag* and *pAct5C-Pro-TEV6HisV5-DNT2-CK*. (4) Co-IP Kek2-DNT2: *pAct5C-Kek2-3xFlag* and *pAct5C-DNT2-FL-HA*. (5) Co-IP Kek3-DNT2: *pAct5C-Kek3-3xFlag* and *pAct5C-DNT2-FL-HA*. (5) Co-IP kek6-Toll-6: *pAct5C-Kek6-HA* and *pAct5C-Toll-6*^*CY*^*-3xFlag*, *pAct5C-Toll-7*^*CY*^*-3xFlag*.(6) Co-IP Kek6-CaMKII: *pAct5-CaMKII-HA* and *pAct-kek-6-FLAG*. (7) Co-IP kek6-VAP33A: *pAct5-Vap33A-HA* and *pAct-kek-6-FLAG*. 48h after transfection cells were harvested and washed in PBS. Final cell pellets were lysed in 600 μL NP-40 buffer (50 mM Tris-HCl pH:8.0, 150 mM NaCl, 1% Igepal-630) supplemented with protease inhibitor cocktail (Pierce). For V5 and HA immuno-precipitations,500 μL of lysates from single or co-transfected cells were incubated with 1 μg of mouse anti-V5 or 1 μg of mouse anti-HA antibodies overnight at 4°C, then the lysate plus antibody mixtures were supplemented with 25 μL of protein-A/G megnetic beads (Pierce) and incubated for 1 h at room temperature. For Flag immunopreciptations, lysates were incubated with anti-Flag antibody conjugated magnetic beads (Sigma-Aldrich) overnight at 4°C or for 2h at room temperature. Beads were washed thoroughly in NP-40 buffer and/or PBS, or in TBST buffer. Proteins were eluted in 40 μL of 2x Laemmli-buffer and analysed by Western blot following standard procedures.

### Pull-down assays and proteomics

S2 cells were transfected with 4 μg *pAct5C-Kek6-3xFlag* expression construct. For controls mock-transfected cells (i.e. no construct) were used. After transfection cells were processed as described above for co-immunoprecipitation. 600 μL of cell lysates were incubated with 20 μL of anti-Flag conjugated magnetic beads overnight at 4°C. Beads were washed thoroughly in PBS, then proteins were eluted in 40 μL 2x Laemmli-buffer for 10 min at room temperature. Eluted proteins were loaded onto 10% polyacryalamide gels. Alternatively, proteins were not eluted after overnight incubation with anti-Flag beads, but they were re-incubated with whole fly (OregonR) head lysates. Here, 60 heads were lysed in 600 μL of NP-40 buffer. After overnight incubation at 4°C, proteins were eluted and analysed as for S2 cell lysate proteins. Thus, eluted proteins were loaded onto 10% polyacryalamide gels, and the gels were stained with Coomassie Blue and cut into several pieces. Gel pieces were subjected to in-gel digestion with trypsin using a standard protocol. Tryptic peptides were analysed by LC-MS/MS using Ultimate 3000 HPLC coupled to a LTQ Orbitrap Velos ETD mass-spectrometer. Peptide separation, mass spectrometric analysis and database search were carried out as specified at the University of Birmingham Proteomics Facility. Candidate binding proteins were identified on these criteria: (1) Proteins were accepted only if they were identified with at least two high confident peptides. (2) Mock-transfected controls and Kek6-3xFlag samples were compared, and only proteins identified from Kek6-3xFlag lysates or heads, but absent form controls, were considered as possible interacting partners for Kek6.

### Western blotting

Western blot was carried out following standard procedures using antibodies listed here: mouse anti-V5 (1:5000, Invitrogen), mouse anti-HA (1:2000, Roche), chicken anti-HA (1:2000, Aves), rabbit anti-Flag (1:2000, Sigma-Aldrich), HRP-conjugated anti-mouse IgG (1:5000–1:10000, Vector), HRP-conjugated anti-chicken IgG (1:5000, Jackson Immunoresearch), HRP-conjugated anti-rabbit IgG (1:1000–1:5000, Vector), mouse dCaMKII (Cosmo CAC-TNL-001-CAM 1:1000), rabbit α-p-CaMKIIα (Thr 286) (Santa Cruz sc-12886-R 1:1000), mouse Tubulin DM1A (Abcam ab7291 1:10000).

### NMJ dissections and preparation

NMJ preparations were carried out according to [67]. For GFP stainings in *MhcGAL4>UAS-DNT2-FL-GFP*, L3 larvae were placed in agar plates (2%) and left at 29°C for 90 minutes to potentiate the NMJ before dissections [68]. A hundred and twenty female flies and 50 males were placed in a cage with a removable agar and grape juice plate. On the second day, plates were changed for new ones in the morning and evening, and discarded. On the third day, the plates were changed every 1h 30min and discarded. On the fourth day, the plates were changed every 1h 30min and kept in a 25°C incubator for larvae collection. The next day, hatched L1 larvae from each plate were transferred to a vial, and exactly 40 larvae were placed in each vial. When larvae reached L3 stage (5 days after egg laying), they were dissected in low calcium saline as previously described[67], and fixed for 10 minutes in Bouin’s solution (HT10132 SIGMA). Samples were washed 6 times for 10 minutes in PBT (0.1% of Triton in 1M PBS) to remove fixative solution and kept overnight in blocking solution (10% normal goat serum in 0.1% Triton in PBS 1M). Primary antibodies were incubated overnight at 4°C and samples were washed the following day 8 times for 10 minutes in PBT. Secondary antibodies were incubated for 2h at room temperature and samples were washed 8 times for 10 minutes in PBT. Samples were mounted in Vectashield anti-bleacing medium (Vector Labs) in No.1 coverslips. NMJs were analysed for muscle 6/7 only; segments A3 and A4, left and right, were analysed in each larva.

### Immuno-stainings and in situ hybridisations

Antibody stainings in embryos and S2 cells were carried out following standard procedures, using the following primary antibodies at the indicated dilutions: mouse anti-FasII at 1:5 (ID4, Developmental Studies Hybridoma Bank, Iowa); rabbit anti-GFP 1:1000 (Molecular Probes); rabbit anti-GFP 1:4,000 (abcam ab290); mouse anti-Dlg 1:20 (4F3, Developmental Studies Hybridoma Bank, Iowa); rabbit anti-HRP 1:250 (Jackson Immunoresearch); mouse anti-Brp 1:100 (nc82, Developmental Studies Hybridoma Bank, Iowa); rabbit anti-p-CaMKII^T286^ 1:150 (Santa Cruz), raised against mammalian pCaMKII^T286^ detects phosphorylation at Thr287 in Drosophila, the constitutively active form; mouse plain anti-Synapsin at 1:25 (DSHB 3C11). Secondary antibodies were: anti-guinea pig-Alexa 488 at 1:250 (Molecular Probes); biotynilated anti-mouse at 1:300 (Jackson Labs) followed by the ABC Elite Kit (Vector Labs); biotynilated anti-guinea pig at 1:300 (Jackson Labs) followed by Streptavidin-Alexa-488 at 1:400 (Molecular Probes); anti-rabbit-Alexa 488 at 1:250 (Molecular Probes); anti-mouse-Alexa 488 1:250 (Molecular Probes); anti-rabbit-Alexa 647 at 1:250 (Molecular Probes); anti-mouse-Alexa 647 1:250 (Molecular Probes).

In situ hybridizations were carried out following standard procedures, using antisense mRNA probes from 5’ linearised plasmids and transcribed as follows: *lambik*: a 551 nucleotides fragment was cloned into pDONR with primers: forward GGGGACAAGTTTGTAC AAA AAA GCA GGC TAG AAA CTA CGC ATG AGC CTG and reverse GGGGACCAC TTT GTA CAA GAA AGC TGG GTA CCG CTC AAA TGT CCA CTG T; then linearised with HpaI and transcribed with T7 RNA polymerase. *CG15744*: a 569 nucleotides fragment was cloned into pDONR with primers: forward GGGGACAAGTTTGTAC AAA AAA GCA GGC TGG ATT GGA TAG CCT TGG TGA and reverse GGGGACCAC TTT GTA CAA GAA AGC TGG GTT TCG CTT CCA TCT CCA TCT C (linearised with HpaI, transcribed with T7); *CG16974*: a 548 nucleotides fragment was cloned into pDONR with primers: forward GGGGACAAGTTTGTAC AAA AAA GCA GGC TTA TAT GAA TCC CGA AGG CGC and reverse GGGGACCAC TTT GTA CAA GAA AGC TGG GTT TGG GGG GAG TAG ATG GTA A (linearised with HPA1, transcribed with T7); *kek1* (*SD01674*+*pOT2* cDNA clone; linearised with EcoRI, transcribed with SP6 RNA polymerase); *kek2* (*NB7*+*pNB40*; HindIII; T7); *kek3* (HpaI; T7); *kek4* (*GH27420*+*pOT2*; EcoRI; SP6); *kek6* (in *pDONR*, HpaI; T7); *CG15744* (in pDNOR, HpaI; T7); *CG16974* (in pDNOR, HpaI; T7); *lambik* (in pDONR, HpaI; T7). Colorimetric reaction was using Alkaline Phosphatase conjugated anti-DIG conjugated.

### Microscopy and imaging

Wide-field Nomarski optics images were taken with a Zeiss Axioplan microscope, 63x lens and JVC 3CCD camera and Image Grabber graffics card (Neotech) and a Zeiss AxioCam HRc camera and Zen software. Laser scanning confocal microscopy was carried out using inverted Leica SP2 AOBS, upright Leica SP8 or inverted Zeiss LSM710 laser scanning confocal microscopes, with 40x lens, 1024 x 1024 resolution, 0.25μm step for anti-Brp, and 0.5 μm step for the rest. Images were compiled using ImageJ, Adobe Photoshop and Illustrator.

For NMJ data, all images provided in the figures are projections from Z stacks; all the quantitative anlyses were carried out in the raw stacks, not in projections. Muscle surface area was measured from bright field images using ImageJ. Anti-HRP was used to measure total terminal axonal length using ImageJ, and branching points. Boutons were visualized with anti-Dlg, and total boutons as well as separately Is and Ib boutons, were counted manually with the aid of the ImageJ Cell Counter plug-in. Automatic quantification of anti-Brp and anti-pCaMKII^T286^ were carried out in 3D throughout the stacks of images using the DeadEasy Synapse ImageJ plug-in that we previously validated [39], and anti-Synapsin was analysed with a slightly modified version.

**DeadEasy Synapse** has already been described and validated [39]. DeadEasy Synapse first reduces the Poisson noise, characteristic of confocal microscopy images, of each slice in the stack using a median filter. Subsequently, in order to separate signal (e.g. Brp+ active zones) from the background, images are segmented. Since the intensity of the staining varied within each image and from image to image, the maximum entropy threshold method [69] was used to find a local optimal threshold value for each pixel. For this, we used a square window of size 15x15, sufficient to find the local optimal threshold around each pixel in an image. In this way, each pixel is considered part of an active zone if the value of the pixel is higher than the local threshold, otherwise assigned to the background. Since this method is computationally expensive and very low intensity pixels correspond to background, it was possible to reduce the computation time by assigning pixels whose intensity was lower or equal than 20 directly as part of the background and only applying the thresholding method to pixels whose intensity was higher than 20. Finally the volume of the active zones is measured. This method worked just as accurately with anti-Brp, anti-pCaMKII^T286^ and anti-Synapsin stainings. Data were normalized to muscle surface area or axonal length.

### Locomotion assays

L3 wandering larvae were placed one at a time on an agar plate (2%) and left it crawl for 40 seconds. Larvae were filmed crawling across the agar plate and then discarded. Plates were cleared before placing another larva. At least 50 larvae were filmed per genotype, and the test was always done in the morning. Films of 400 frames per larva were analysed using FlyTracker software developed in our lab to obtain the trajectory and speed, as previously described[38].

### Statistical analysis

Data were analysed in SPSS Statistics 21 (IBM) and GraphPad Prism 6. Confidence interval was 95% (p<0.05). Categorical data were tested using χ^2^, and a Bonferroni correction was applied for multiple comparisons. Continuous data were first tested for normality by determining the kurtosis and skewness, and a Levene’s Test was applied to test for homogeneity of variance. Data were considered not normally distributed if absolute kurtosis and skewness values for each genotype were greater than 1.96 x standard error of kurtosis/skewness. Variance of the populations of different samples were considered unequal if Levene’s test for homogeneity of variance gave a p value of <0.05. If samples were normally distributed and variances were equal, Student-t tests were applied for 2-sample type graphs, and One-Way ANOVA was used to compare means from >2 samples. When data were normally distributed, but variances were unequal, Welch ANOVA was used instead. Multiple comparison corrections to normal data were applied using a post-hoc Dunnett test of comparisons to a control, Games-Howell or Bonferroni post-hoc comparing all samples. Non-parametric continuous data were compared using a Mann-Whitney U-test when 2 sample types were being analysed, and a Kruskal-Wallis test for >2 samples, and multiple comparison corrections were applied using a post-hoc Dunn test. See S1 Table for all statistical sample sizes, applied tests and p values.

## Supporting information

S1 Fig Kek-6 is expressed is expressed in Eve+ neurons but not in glia. Confocal images of the VNC neuropile of third instar Kek-6^MIMIC-GFP^ larvae, stained with anti-GFP and (A) anti-Eve and (B) the pan-glial nuclear marker anti-Repo. (A) Eve colocalises with GFP in many cells (arrows indicate examples). Interestingly, most Eve+ neurons are also Toll-6+ (see [38]). (B) Repo does not colocalise with Kek-6^MIMIC-^GFP in any cells, arrows point to examples of Repo+, GFP-negative cells.
(TIF)

S2 Fig *kek6*^*34*^, *kek6*^*35*^ and DNT2^37^ are null mutant alleles. **(A)** *kek6*^*34*^ and *kek6*^*35*^ null mutant alleles are deletions lacking the entire coding region for *kek-6*. (B) Following mutagenesis, these alleles were identified by two-sided PCR. (C) The deletion alleles were confirmed by demonstrating the lack of genomic regions internal to the deletion site. (D) The *DNT2*^*37*^ null mutant allele bears a deletion with a breakpoint at the site of PBac{RB}spz5^e03444^. It lacks the regulatory region, all 5’UTR, the start site, and the first two exons. (E) Reverse Transcription PCR (RT-PCR) using primers to test if *DNT2* mRNA is transcribed downstream of the breakpoint, which should result in a 464bp band. No transcripts were was detected (arrows). See also Fig 2D in[36] where we provided evidence that a construct generated from the sequences downstream of the breakpoint to the terminal stop codon, tagged at the 3’ with HA, did not translate into a protein product in transfected S2 cells. Together, these data demonstrate that *DNT2*^*37*^ is a null allele.
(TIF)

S3 Fig Altered *kek-6* function affects motoraxon targeting at embryonic NMJ. The motoneuron marker FasII reveals motoraxon targeting phenotypes at muscle 6,7,12,13 in stage 17 embryos, in *kek-6* mutants and upon over-expression of *kek-6* in all neurons (with *elavGAL4*). Arrows indicate stereotypic projections in wild-type, and mistargeting in other genotypes. Chi-square p<0.0001 and ***p<0.001 Boferroni corrections, see S1 Table. N = 328–407 hemisegments.
(TIF)

S4 Fig *kek-6* over-expression induces ghost boutons. (A,B) Over-expression of *kek-6* in motoneurons (MN) with *D42GAL4* did not affect bouton number (Dlg, Mann-Whitney U-test not significant). (C-E) Over-expression of *kek-6* induced pre-synaptic ghost boutons lacking a post-synaptic component (arrows: HRP+, presynaptic and Dlg-negative, post-synaptic), (D) higher magnification; (E) quantification. Both bouton number and area increased, albeit not significantly. Mann-Whitney U-tests. See S1 Table. N = 14–66 hemisegments. *Genotypes*: Controls: *y w/+*; *D42GAL4/+*. *kek-6*^*–/–*^: *kek6*^*34*^*/Df(3R)6361; MN>kek6*: *D42GAL4>UAS-kek6-RFP* (with one or two copies of *UAS-kek-6*).
(TIF)

S5 Fig Loss and gain of CaMKII function affect the NMJ. (A) Confocal images of muscle 6/7 NMJs, in A3-4, labeled with anti-HRP for the pre-synaptic terminal, anti-Brp for active zones, anti-Dlg for post-synaptic boutons and anti-pCaMKII^T287^ for the constitutively active form. (B,C) Quantification. (A,B) Inhibiting CaMKII function with Ala in motoneurons (*D42GAL4>UASAla*) decreased NMJ terminal axonal length (HRP,t-test), and active zones. Student t-tests, p<0.005, **p<0.01. (A,C) Pre-synaptic over-expression of constitutively active CaMKII (*D42GAL4>UASCaMKII*^*T287D*^) increased pCaMKII levels (pCaMKII^T287^, Student t-test **p<0.001), axonal length (HRP, Student t-test **p<0.001), Ib bouton number (Dlg, Mann-Whitney U-test ***p<0.001), and active zones (Brp, Mann-Whitney U-test **p<0.01). See S1 Table. N = 25–66 hemisegments.
(TIF)

S1 Table Statistical analysis details. For all genotypes, sample sizes, tests and p values, see this table.
(XLSX)

S2 Table Kek6 pull-down from S2 cells. List of S2 cell lysate proteins binding Kek6-Flag, but not mock-transfection controls, and detected with more than one peptide.
(XLSX)

S3 Table Kek6 pull down from adult fly heads. List of adult fly head proteins binding Kek6-Flag, but not mock controls, and detected with more than one peptide.
(XLSX)
