# Supplementary figures and images for: Kek-6: A truncated-Trk-like receptor for Drosophila neurotrophin 2 regulates structural synaptic plasticity
Source: PLoS Genet. 2017 Aug 28;13(8):e1006968. doi: 10.1371/journal.pgen.1006968 (PMC5591008; doi:10.1371/journal.pgen.1006968)

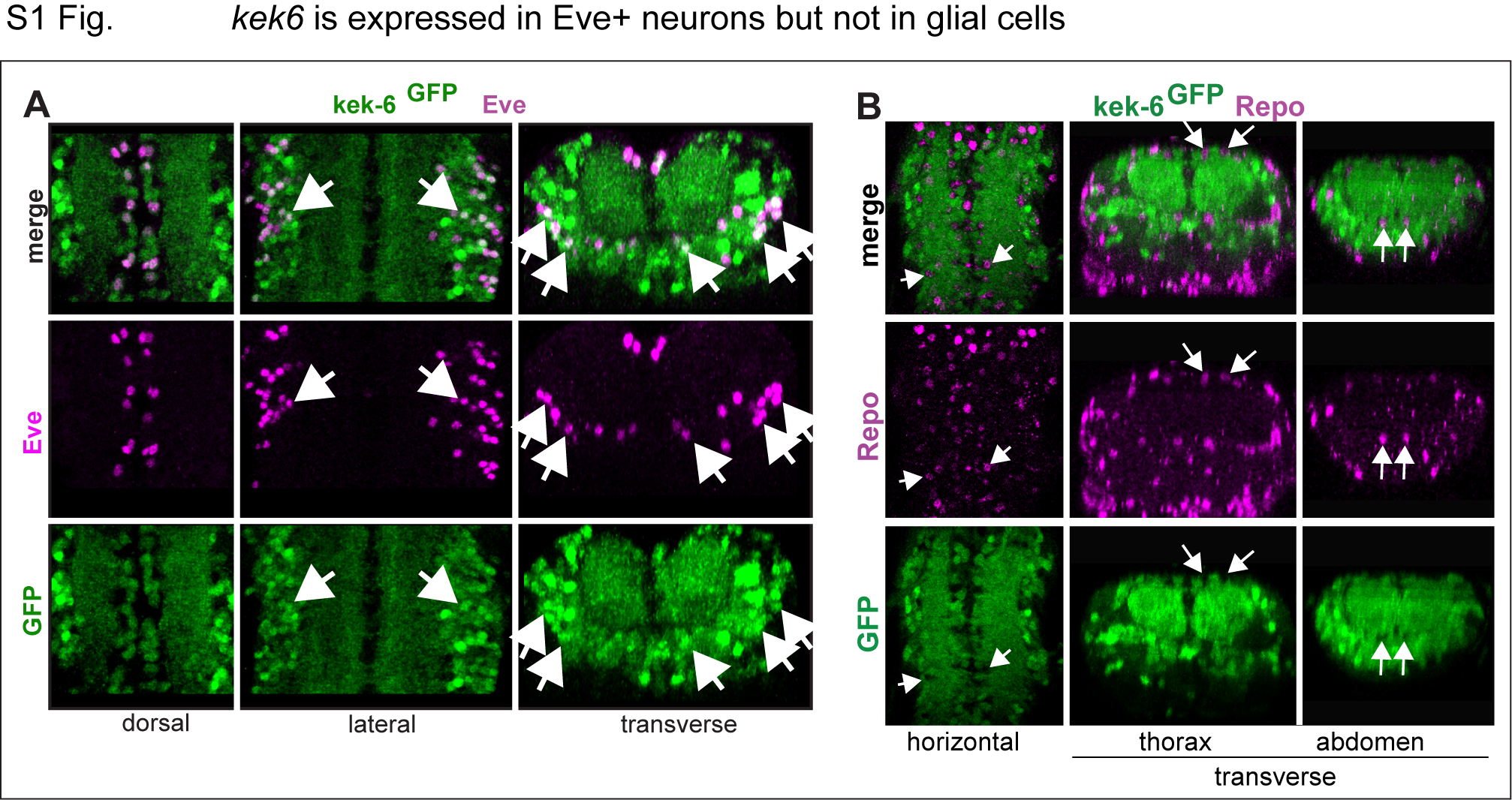

Supplement: S1 Fig — Confocal images of the VNC neuropile of third instar Kek-6MIMIC-GFP larvae, stained with anti-GFP and (A) anti-Eve and (B) the pan-glial nuclear marker anti-Repo. (A) Eve colocalises with GFP in many cells (arrows indicate examples). Interestingly, most Eve+ neurons are also Toll-6+ (see [38]). (B) Repo does not colocalise with Kek-6MIMIC-GFP in any cells, arrows point to examples of Repo+, GFP-negative cells. (TIF) [file pgen.1006968.s001.tif]

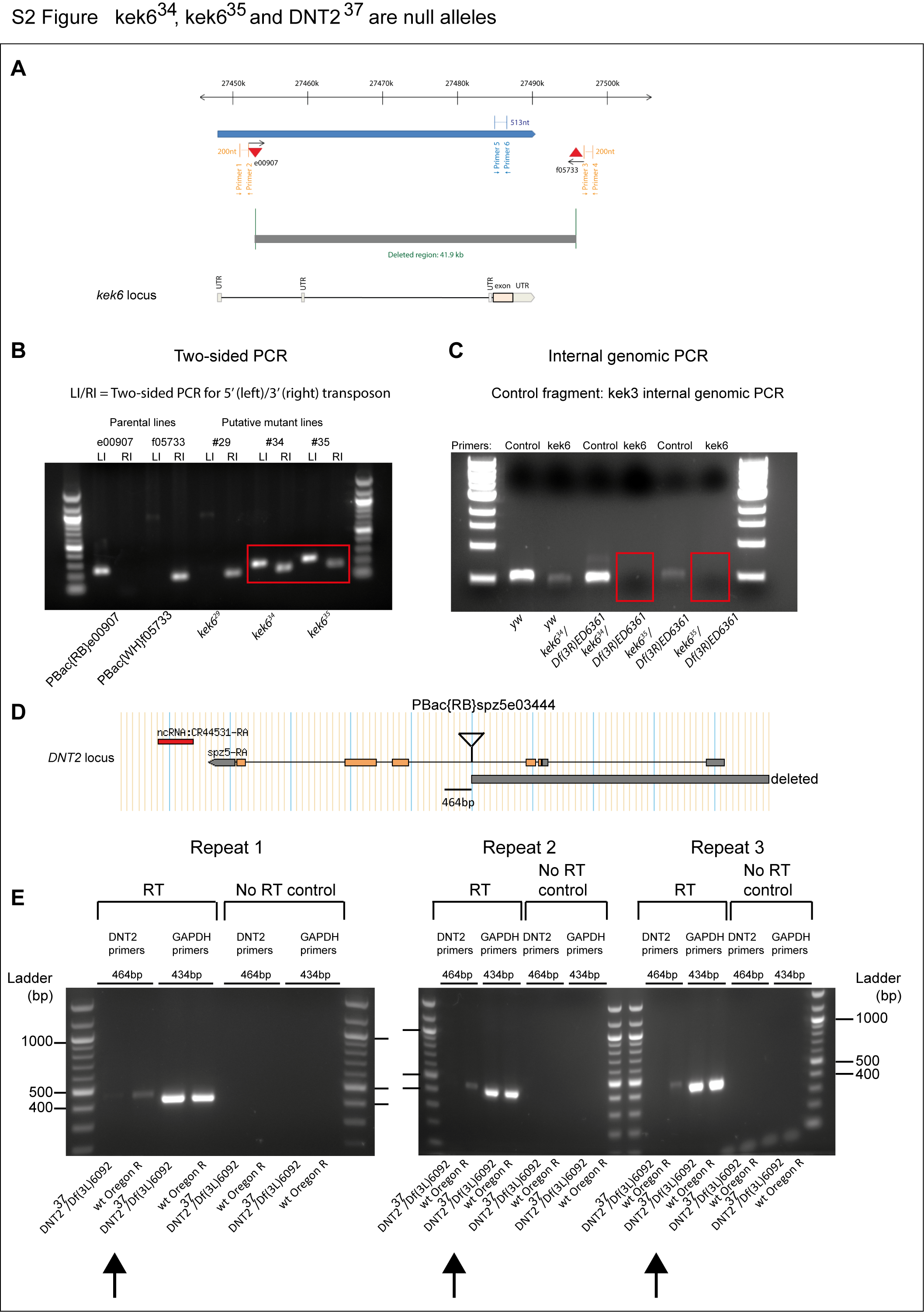

Supplement: S2 Fig — (A) kek634 and kek635 null mutant alleles are deletions lacking the entire coding region for kek-6. (B) Following mutagenesis, these alleles were identified by two-sided PCR. (C) The deletion alleles were confirmed by demonstrating the lack of genomic regions internal to the deletion site. (D) The DNT237 null mutant allele bears a deletion with a breakpoint at the site of PBac{RB}spz5e03444. It lacks the regulatory region, all 5’UTR, the start site, and the first two exons. (E) Reverse Transcription PCR (RT-PCR) using primers to test if DNT2 mRNA is transcribed downstream of the breakpoint, which should result in a 464bp band. No transcripts were was detected (arrows). See also Fig 2D in[36] where we provided evidence that a construct generated from the sequences downstream of the breakpoint to the terminal stop codon, tagged at the 3’ with HA, did not translate into a protein product in transfected S2 cells. Together, these data demonstrate that DNT237 is a null allele. (TIF) [file pgen.1006968.s002.tif]

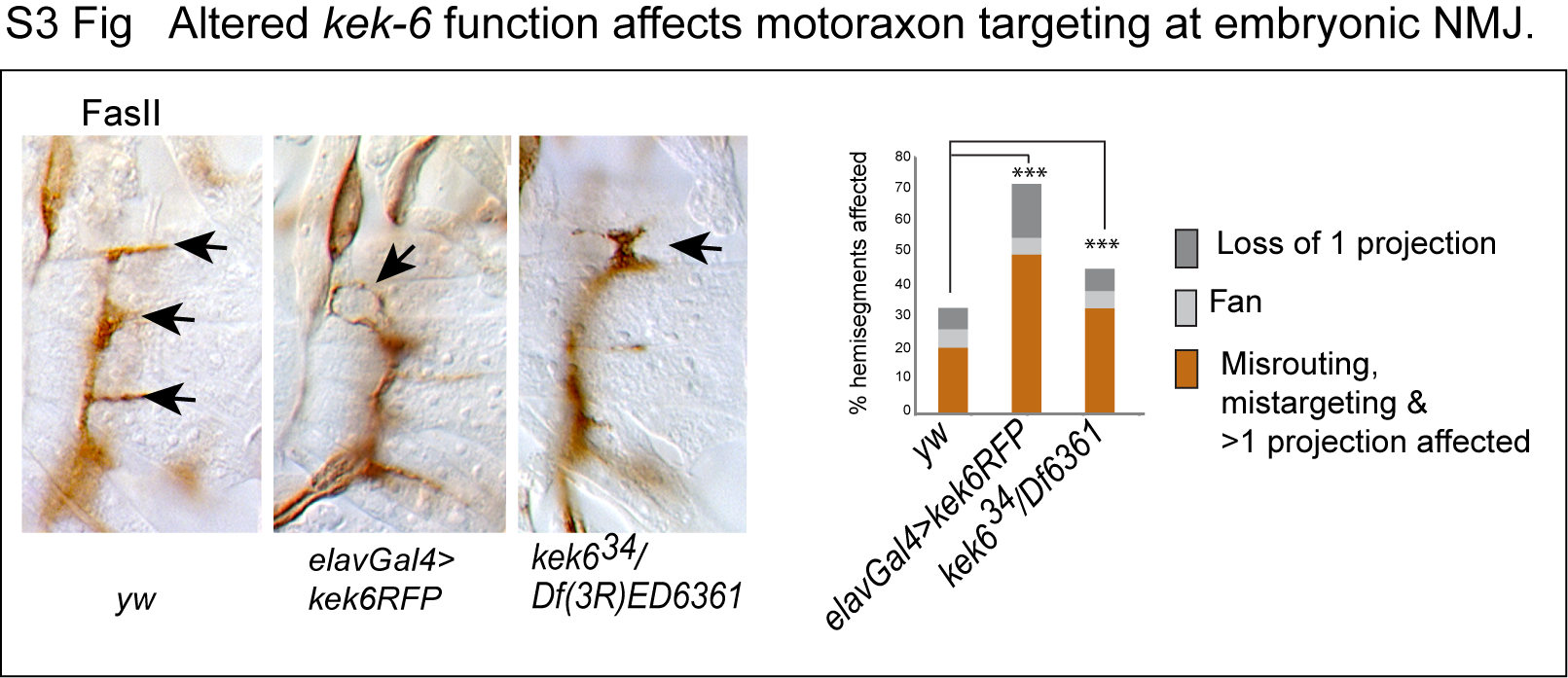

Supplement: S3 Fig — The motoneuron marker FasII reveals motoraxon targeting phenotypes at muscle 6,7,12,13 in stage 17 embryos, in kek-6 mutants and upon over-expression of kek-6 in all neurons (with elavGAL4). Arrows indicate stereotypic projections in wild-type, and mistargeting in other genotypes. Chi-square p<0.0001 and ***p<0.001 Boferroni corrections, see S1 Table. N = 328–407 hemisegments. (TIF) [file pgen.1006968.s003.tif]

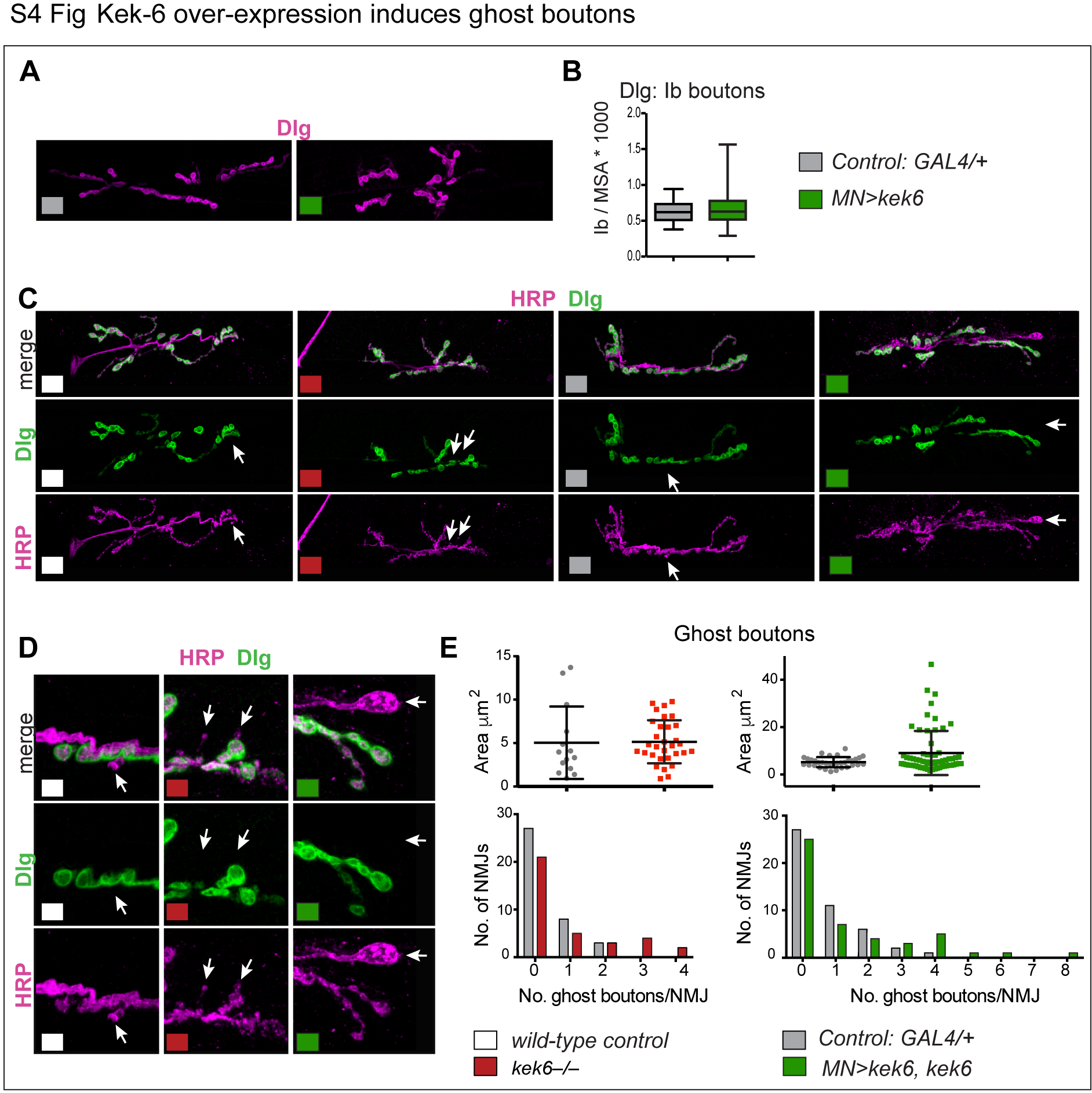

Supplement: S4 Fig — (A,B) Over-expression of kek-6 in motoneurons (MN) with D42GAL4 did not affect bouton number (Dlg, Mann-Whitney U-test not significant). (C-E) Over-expression of kek-6 induced pre-synaptic ghost boutons lacking a post-synaptic component (arrows: HRP+, presynaptic and Dlg-negative, post-synaptic), (D) higher magnification; (E) quantification. Both bouton number and area increased, albeit not significantly. Mann-Whitney U-tests. See S1 Table. N = 14–66 hemisegments. Genotypes: Controls: y w/+; D42GAL4/+. kek-6–/–: kek634/Df(3R)6361; MN>kek6: D42GAL4>UAS-kek6-RFP (with one or two copies of UAS-kek-6). (TIF) [file pgen.1006968.s004.tif]

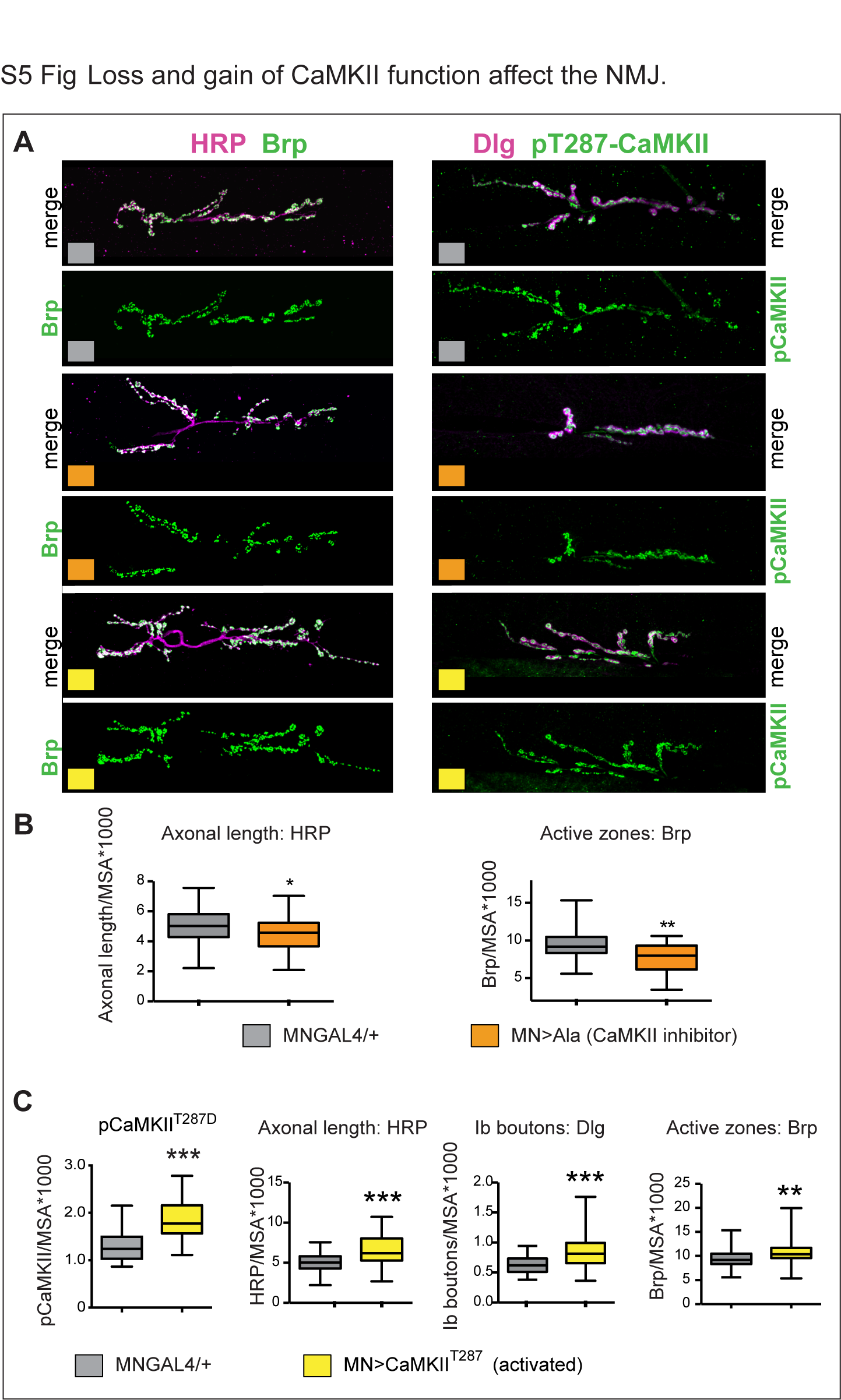

Supplement: S5 Fig — (A) Confocal images of muscle 6/7 NMJs, in A3-4, labeled with anti-HRP for the pre-synaptic terminal, anti-Brp for active zones, anti-Dlg for post-synaptic boutons and anti-pCaMKIIT287 for the constitutively active form. (B,C) Quantification. (A,B) Inhibiting CaMKII function with Ala in motoneurons (D42GAL4>UASAla) decreased NMJ terminal axonal length (HRP,t-test), and active zones. Student t-tests, p<0.005, **p<0.01. (A,C) Pre-synaptic over-expression of constitutively active CaMKII (D42GAL4>UASCaMKIIT287D) increased pCaMKII levels (pCaMKIIT287, Student t-test **p<0.001), axonal length (HRP, Student t-test **p<0.001), Ib bouton number (Dlg, Mann-Whitney U-test ***p<0.001), and active zones (Brp, Mann-Whitney U-test **p<0.01). See S1 Table. N = 25–66 hemisegments. (TIF) [file pgen.1006968.s005.tif]
